# A novel framework for TER allocation using multilayer perceptron and intuitionistic fuzzy Z numbers for talent management

**DOI:** 10.1038/s41598-025-15055-z

**Published:** 2025-08-26

**Authors:** Tingting Yang

**Affiliations:** Publicity Department, Changsha Normal University, Changsha, Hunan 410100 China

**Keywords:** Training and education resources allocation, Multi-layer perceptron, Talent Management, Intuitionistic Fuzzy Z Numbers, Fuzzy logic, Biotechnology, Engineering

## Abstract

The effective allocation of Training and Education Resources (TER) is one of many organizational pathways to maximizing workforce capability and employee development. Conventional means of assessing employees and allocating employee resources are inadequate in managing uncertainty, imprecision, or performance data in complex forms and paradigms. In this paper, a new model is proposed that implements an integrated application of Intuitionistic Fuzzy Z-Numbers and multi-layer perceptron networks for a more realistic and accurate employee performance evaluation and resource allocation. The proposed model employs fuzzy logic to handle uncertainty in performance evaluation, such as degrees of membership, non-membership, and hesitancy. The multi-layer perceptron network predicts employee performance trends to help allocate resources, if required, while performance is progressing. The model was analyzed through experimental analysis, with a significant R^2^ factor value (0.9967). The R^2^ proves that the model predicts performance and improves employee resource allocation distribution. The proposed model is a demonstrative improvement in employee performance evaluation tools, compared to traditional frameworks of evaluation and allocation. The model is flexible enough to help organizations conduct effective talent management and allocate resources, with a handling degree of uncertainty, when their available employee performance data is incomplete. However, this framework should be explored further in terms of effective models that reduce data sparsity as well as real-time integrations and adjustments. Ultimately, this research presents an adapted and scalable model of organizational talent management and organizational performance.

## Introduction

TER allocation is critical in talent management, especially in dynamic working contexts where workforce demand and skills developed are dynamic and changing. Conventional allocation techniques have difficulties dealing with the uncertainty and vagueness present in human-centric decision-making, and they may finally result in suboptimal resource usage^1^. Solving these challenges demands comprehensive computational models that can adopt temporal aspects and dealing with inexact and uncertain information.

LSTM is an important class of recurrent neural networks (RNN) that have become very popular because of their effectiveness in time-series predictions and sequence-based applications. These networks are capable of capturing long-term dependencies within data. In educational domains, they can successfully predict individual students’ performance or skill development, while enabling personalized learning environments^2,3^. By considering all historical data as sequential patterns, and a student’s current needs, LSTMs can help predict the future trajectory, allowing an educational institution to assign resources more effectively or design training programs that meet the needs of the students or employees for whom they are responsible^4^. A persistent problem for researchers and organizations as they try to characterize educational sentiment in the field, is incorporating the uncertainty with respect to human behavior and decision making. This is particularly relevant in talent management or educational domains.

Henceforth, fuzzy logic approaches are to be considered, especially the use of intuitionistic fuzzy sets and extending them with Intuitionistic Fuzzy Z$\check{\phantom {0}}$ Numbers, to develop integrated LSTM models. These fuzzy approaches recognize degrees of membership, non-membership, and hesitation, which provide more nuanced representations of uncertainty and subjective evaluations in talent management (Z-numbers and the applications in fuzzy systems, Interval Intuitionistic Fuzzy Decision Model with Abnormal Information and Its Application in Talent Selection)(March, 2020)^5,6^. This allows for integrating both quantitative temporal trends, applying LSTM models and the qualitative uncertainties present in qualitative evaluations^7,8^.

Recent research has shown the ability of hybrid models utilizing LSTM in combination with intuitionistic fuzzy logic. For instance, The Sequential Intuitionistic Fuzzy (SIF) LSTM model attained high accuracy in assessing factors influencing the quality of teaching provided by universities. This was an excellent demonstration of the potential effectiveness of this approach to aid in educational resource allocation and evaluation of influencing factors of China university teaching quality using SIF-LSTM model^9^. Fuzzy-enhanced LSTM modelling using an architecture is more robust and interpretable in environments of decision uncertainty an empirical evaluation of fuzzy bidirectional long short-term memory with a soft computing-based decision-making model^10^. The framework of Multi-Attribute Group Decision Making (MAGDM) has been proposed by Niu et al.^11^ with a purpose of Spherical Fuzzy Z-Numbers (SF ZNS), which helps manage uncertainties better than fuzzy sets. The CRADIAS method permits the ranking of alternative actions when the weights of the attributes are not known, which uses the CRITIC technique to identify the attribute weights. The research undertaken discusses the theoretical basis of SF Z-numbers and their implementation in the MAGDM process, while a comparison with the MARCOS method confirms their validity in group decision-making contexts. Wu et al.^12^ introduced the concept of Spherical Z-Numbers (SZN) in multi-attribute group decision-making (MAGDM), addressing the limitations of traditional fuzzy sets by incorporating both membership and reliability factors. Unlike conventional fuzzy sets, SZN provides a more robust framework for handling uncertainty in decision-making processes. Their model not only considers expert evaluation reliability but also integrates prospect theory to account for decision-makers’ psychological behaviors, such as risk aversion.

Building on these advances, this paper proposes a novel framework that integrates multi-layer perceptron networks with Intuitionistic Fuzzy Z$\check{\phantom {0}}$ Numbers to optimize training and education resource allocation within talent management. The proposed approach aims to improve allocation accuracy and flexibility by effectively managing temporal dynamics and uncertainty, thereby supporting more adaptive and personalized talent development strategies.

### Motivation

The following points highlight the primary reasons for adopting this method and its potential benefits in addressing existing challenges in performance evaluation and resource allocation.

- Traditional methods of employee performance evaluation often struggle with uncertainty and imprecision in human judgment, leading to unreliable resource allocation.
- Existing systems tend to allocate Training and Education Resources (TER) suboptimally due to their inability to handle dynamic and evolving employee performance data effectively.
- Conventional performance evaluation methods fail to capture the complexity and subjectivity inherent in human assessments; this work aims to enhance objectivity and precision.
- The use of machine learning, specifically MLP networks, provides a method for predicting employee performance trends, allowing organizations to allocate resources proactively.
- By integrating IFZN, this model better represents uncertainty, hesitation, and subjective judgment in performance evaluations.
- The framework offers a scalable, flexible approach to talent management, ensuring efficient resource distribution based on more accurate and detailed performance assessments.

### Research contributions

- This study presents a unique method by combining Intuitionistic Fuzzy Z-Numbers (IFZN) with multi-layer perceptron methods, to further address uncertainty and complexity in HR analytics.
- The framework utilizes fuzzy logic to generate detailed performance metrics, including membership, non-membership, and hesitation degrees while leveraging multi-layer perceptron for accurate predictive modeling and analysis.
- A data-driven approach is employed to ensure resource allocation, including required hours of training, mentorship, and associated expenses, is made efficiently and strategically aligned with the organization’s goals.
- At 100% data integrity via painstaking preprocessing, including missing data and normalization, all of which guarantee reliable and accurate analytical outcomes.
- Scalability and High-Performance Execution: The model can efficiently process large datasets and achieves exceptional performance with a 99.9% correlation in fuzzy logic and deep learning results (R$^{2}$ = 0.9967 for prediction accuracy for 177,417 employees).

### Paper organization

This paper is organized as follows: The Introduction outlines the motivation for the work and the need for better resource allocation methods. The Related Work reviews existing literature on employee performance evaluation and resource allocation. The Proposed Model presents the methodology, combining Intuitionistic Fuzzy Z-Numbers with Multi-Layer Perceptron networks to address uncertainty. The Data Collection and Preprocessing section explains the dataset and preprocessing steps. The Experimental Results and Evaluation showcase the model’s effectiveness with visualizations. The Discussions cover the model’s strengths, limitations, and future research directions. Finally, the Conclusion summarizes the findings and contributions, emphasizing the potential for dynamic resource allocation improvements.

## Related work

Human resource (HR) productivity is one of the primary focal points of all organizational development considerations; it is a crucial aspect of overall performance. Azizi et al.^13^ explored the concept of sustainable productivity in a railway operation company and revealed significant elements potentially influencing HR productivity. Among these factors were the HRM policies and practices, employees’ motivation or will to work, and people contributing towards public welfare. They also noted the regard paid to organizational culture, leadership styles, incentive structures, and ergonomic conditions were impacting HR productivity.

Likewise, Harati Mokhtari and Younespoor^14^ examined HR productivity of Chabahar Port, they employed the analytical hierarchy process (AHP) to rank influencing factors of greater significance. The authors deemed leadership style as the most impactful factor, followed by job-person fit, competency-based promotion system, and work ethic. Secondary factors of HR productivity included wage system, training, and compliance regulation. Oyefusi et al.^15^ examined the individual and impact of team and group dynamics on organizational performance, focusing on the effect of leadership behaviors, employee satisfaction, and organizational communication, particularly for a heterogeneous workforce comprising new job-starters or recently recruited employees, on aspects of productivity. The authors also emphasized leadership personality dimensions on organizational behavior and, by extension, its impact on employee performance.

Delbari et al.^16^ examined key productivity determinants of staff including both individual and organizational factors at Azad University and the University of Qom. delbari et al. used interviews and surveys to identify the goal setting, training, human relationships, organizational culture, and job structure had a positive impact on productivity, they also identified that individual factors received higher ratings compared to organizational factors, which highlighted the personal attributes and development over the other determinants. More generally, HR productivity is closely linked to how well employees are supported in their roles. Positive human resource procedures, for instance, strategic recruitment, in-role training, soft skill development, incentives, and well-being, significantly relate to employee engagement and performance^17^, which in turn contributes to increased employee motivation and productivity^18^.

To more effectively deal with the complexities and ambiguities in HR systems, fuzzy logic appears to be a robust modeling tool. It allows for nonlinear relationships to be represented, allows for linguistic expert input to be integrated, and also allows for quantitative and qualitative input to be merged - fuzzy set-theoretic modeling is fundamentally well-suited to multifaceted decision-making environments^19^. Fuzzy set design methods, for instance fuzzy rating scales using questionnaires, have also found widespread application to identify subjective human input using linguistic variables^20^. The use of fuzzy logic in HR management has been developing in recent years. Demirel and Çubukçu^21^ used a fuzzy decision model with Mamdani’s approach to make recruitment more objective by evaluating candidate qualifications. Zhang et al.^22^ used a fuzzy comprehensive evaluation and AHP to optimize organizational human resource structure, using numerical data together with expert opinion to help make decisions. More recently, the Fuzzy Optimized Talent Cultivation Engine (FOTCE) was presented that combines ANFIS with particle swarm optimization to conduct performance and engagement tracking.

Many studies have addressed fuzzy logic in tandem with talent evolution. Lin et al.^23^ examined competencies for employees in the hospitality industry with a fuzzy-assisted decision model based on attention analysis. This same study verified growth by determining attitude and capability, but noted limitations because it was a non-technological industry. Lin et al.^24^ investigated training programs sponsored by the government in Taiwan’s hospitality industry. The participants had an 88% effectiveness rate for the training with 404 participants. Training satisfaction and skills application were recognized as goals but the research indicated that other environmental and individualized factors outside of the research may affect these results.

Yu et al.^25^ used fuzzy hierarchy (FH)-based models with AHP and entropy analysis to create a model for evaluating talents of students in a training context. They achieved a reasonable level of predictive accuracy (MSE = 0.428, RMSE = 0.448, R^2^ = 0.66), where the limitation of the research considered the relations with instructors and students. Lun et al.^26^ developed a hybrid evaluation model to evaluate skills for a competition participant using PCA, K-means clustering and particle swarm optimization. The average skill acquisition was 0.312. Again, there were concerns raised about the competitiveness of skills and effectively limiting creativity by regulating the competition based on standardized measures.

Wang et al.^27^ formulated a talent assessment framework using CRITIC and gray correlation analysis combined with the TOPSIS method, delivered as an Input-Process-Output (IPO) model. Their study was based on ten cases in a university context. The findings revealed that aligned practices in moves from education to industry would increase student satisfaction, but that challenges related to the implementation of a training platform were acknowledged.

At the national perspective, Chang et al.^28^ used a performance evaluation model for National Human Resource Development (NHRD), and their focus was on talent cultivation and value creation. By analyzing data from 60 countries, they included time-lagged input-output analysis. They stated the talent use efficiency accounted for 83.3% of variation in performance. The authors also discussed the values embedded in cultures in terms of the laws, socioeconomics, and so on which ultimately have implications for talent development outputs.

Wu et al.^29^ contributed to the literature by combining data mining, fuzzy logic, neural networks, and AI for HR. They were able to use their incremental clustering-based model in HR systems at universities, allowing for performance appraisal, association rule mining, and data visualization. This study highlighted how HR analytics can take advantage of intelligent data processing. Zhao et al.^30^ noted that as market competitiveness shifts from price to talent, it has had implications regarding the importance of strategic HRM. They used fuzzy theory decision making to assess professional and comprehensive indicators in HR systems, and they found performance management and employee engagement to be weak areas assumed within a sound overall HR system.

Tang et al.^31^ focused on how HR can be enhanced in universities using AHP, fuzzy evaluation and educational-database analysis. The study developed greater incentive systems, defined organizational roles, clarified HR systems, and scaffolded job descriptions around the implied outcome strategy; it still encouraged using best practices from outside the organization to improve HR in higher education organizations. Hsieh et al.^32^ were primarily interested in creating a knowledge management (KM) culture to support talent development. Their research combined KM tools, competency management systems, e-learning systems, and training programs to assist organizations in aligning their development strategy with the organization’s mission. They concluded that successful implementation requires not only technical systems but also a cultural foundation that has an appreciation for shared knowledge and manager synergy. Recent advancements in fuzzy inference systems (FIS) have focused on enhancing the expressiveness and interpretability of decision rules by combining fuzzy logic with neural networks. The Z-number based Neural Network Structured Inference System (ZNIS) utilizes Z-numbers, which represent uncertainty by combining fuzzy sets with probability measures, improving decision-making under uncertainty. The system incorporates Differential Evolution with Constraints (DEC) optimization for more efficient training and better rule generation. Compared to traditional Type-1 and Type-2 Fuzzy Logic Systems (FLS), ZNIS offers superior adaptability, transparency, and computational power, making it a promising tool for applications like medical diagnosis and nonlinear system identification^33^. The evaluation and optimization of talent management have evolved with the introduction of advanced algorithms that address the complexities of assessing employee performance and development. One such approach is the Fuzzy Optimized Talent Cultivation Engine (FOTCE), which combines Adaptive Neuro-Fuzzy Inference Systems (ANFIS) and Adaptive Hybrid Particle Swarm Optimization (AHPSO) to evaluate factors like job satisfaction, work-life balance, and career progression. Traditional models often struggle with subjectivity and uncertainty, but FOTCE leverages fuzzy logic to manage these challenges and provides a more comprehensive, data-driven evaluation framework. Unlike models such as IAA-NRM, FAHP, and DEA, which focus on specific factors like performance or satisfaction, FOTCE integrates multiple criteria to offer a more holistic and adaptive approach to talent management, ultimately enhancing organizational competitiveness and employee retention^34^.

## Proposed model

**Fig. 1:**
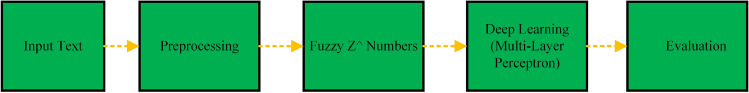
Workflow of the proposed model for TER allocation.

This section sets forth the mathematical methodology used for assessing employee performance and efficiency and the distribution of resources using IFZN, and multi-layer perceptron. The analysis is divided into four major steps, data cleaning, fuzzy evaluation, deep learning augmentation, and resource allocation, as shown in Fig. 1.

### Data collection

The dataset utilized in this study, titled “Employee’s Performance for HR Analytics”, was sourced from Kaggle, a reputable platform for data science and machine learning projects. This dataset comprises employee records from a corporate environment, encompassing various attributes pertinent to human resource analytics.

### Preprocessing

Let the raw dataset be represented as a matrix ${\textbf{D}} = [D_{ij}]$ where each element $D_{ij}$ represents the value of the *j*-th attribute for the *i*-th employee. The dataset consists of *N* rows (employees) and *M* columns (attributes).

#### Handling missing values

For attributes with missing values, the imputation is performed based on the mean or median. Let the missing value $D_{ij} = \emptyset$ and we define the imputed value ${\bar{D}}_{ij}$ as the mean value of the attribute *j*:

1 $$\begin{aligned} {\bar{D}}_{ij} = \frac{1}{n} \sum _{i=1}^{n} D_{ij} \end{aligned}$$

where $n$ is the number of non-missing values for attribute $j$.

This ensures that the dataset is complete and ready for analysis.

#### Normalization

The normalization step transforms the data to a common scale. The formula for normalization of the *j*-th attribute is:

2 $$\begin{aligned} x_{ij}^{\text {norm}} = \frac{x_{ij} - \min (x_j)}{\max (x_j) - \min (x_j)} \end{aligned}$$

where:

- $x_{ij}$ is the value of the *j*-th attribute for employee *i*,
- $\min (x_j)$ and $\max (x_j)$ are the minimum and maximum values of the *j*-th attribute across all employees.

This normalization ensures that all attributes are on the same scale, allowing for fair comparisons and computations.

#### Tokenization

Tokenization is an important step in the preprocessing of textual data. This is important because tokenization is the transformation from the raw text into discrete tokens. In this research project, tokenization is the action of text processing, an example of data preprocessing before the actual analysis of the textual data. Tokenization is simply partitioning a sequence of characters/words into fewer elements that can be utilized by a machine learning algorithm. For instance, consider the sentence:

“Employee performance evaluation is crucial.”

Through tokenization, this sentence is split into individual words (tokens) as:

[“Employee”, “performance”, “evaluation”, “is”, “crucial”]

Every word in a sentence is considered a separate token. This is called word-level tokenization. Sentence-level can be done as well; the text is treated as separate sentences. For instance:

“Employee performance is key. Evaluating performance helps improve productivity.”

After sentence-level tokenization, the output becomes:

[“Employee performance is key.”, “Evaluating performance helps improve productivity.”]

In any case, tokenization takes the unstructured text and converts it into smaller, more manageable units that can be further processed. Tokenization is a mechanism used to split the text into pieces that can be further analyzed or treated as inputs to machine learning algorithms. Once the text has been tokenized, each token can then be processed in a number of different ways, such as one-hot encoding or word embeddings. For example, let us assume the tokenized representation of an employee’s performance review looks like this:

3 $$\begin{aligned} T = [t_1, t_2, t_3, \ldots , t_k] \end{aligned}$$

where *T* is the token set, $t_i$ represents individual tokens, *k* is the count of tokens. These tokens can be embedded into numerical vectors and used as inputs to machine learning models. Upon tokenization of the text, the analysis can continue through a token representation or structure of the data, which may facilitate downstream tasks such as sentiment analysis, feature extraction, and predictions. Therefore, tokenization is a transferable step in preparing textual data for modeling and analysis.

### Intuitionistic fuzzy Z-numbers (IFZN)

In the study, employee performance evaluation is performed using IFZN, enabling representation of uncertainty that exists in HR data. The IFZN framework utilizes a mathematical approach to evaluate employee performance by integrating degree of certainty and uncertainty from membership, non-membership, and hesitation.

Let $P=\{p_1,p_2,\dots ,p_k\}$ represent the performance metrics for each employee *i*, where $p_j$ is the *j*-th performance metric (e.g., previous year rating, training scores, KPIs, awards won). For each performance metric $p_{ij}$, a corresponding weight $w_j$ is assigned to indicate its relative importance in the overall evaluation. We calculate the weighted performance score for employee *i* based on these performance metrics:

4 $$\begin{aligned} p_i^{\text {fuzzy}} = \sum _{j=1}^{k} w_j p_{ij} \end{aligned}$$

where:

- $p_{ij}$ is the value of the *j*-th metric for employee *i*,
- $w_j$ is the weight assigned to the *j*-th metric,
- $p_i^{\text {fuzzy}}$ is the overall fuzzy performance score for employee *i*.

The performance score $p_i^{\text {fuzzy}}$ represents a single value derived from the weighted sum of many performance indicators. Nonetheless, there is uncertainty in evaluating employee performance, which typically involves subjective judgments, and we will apply the IFZN to represent the uncertainty.

An IFZN $Z_i$ for employee *i* is a triplet $(\mu _i, \nu _i, \pi _i)$ where:

- $\mu _i$ is the membership degree, indicating the degree of certainty that the employee’s performance belongs to a particular category (e.g., Good, Average, Poor),
- $\nu _i$ is the non-membership degree, indicating the degree of certainty that the employee does not belong to the performance category,
- $\pi _i$ is the hesitation degree, indicating the uncertainty in deciding whether the employee belongs to the category or not.

The degrees $\mu _i$, $\nu _i$, and $\pi _i$ satisfy the following condition:

5 $$\begin{aligned} \mu _i + \nu _i + \pi _i = 1 \end{aligned}$$

This constraint ensures that the total uncertainty (membership, non-membership, and hesitation) sums up to 1, representing the total possible “fuzzy” uncertainty.

The fuzzy set is defined by the membership function $\mu _i$, which can be computed using the sigmoid function:

6 $$\begin{aligned} \mu _i = \frac{1}{1 + e^{- \left( \sum _{j=1}^{k} w_j p_{ij} \right) }} \end{aligned}$$

This sigmoid function maps the aggregated performance score $\sum _{j=1}^{k} w_j p_{ij}$ into a value between 0 and 1, representing the degree of membership. The larger the aggregated performance score, the higher the membership degree, indicating a higher probability that the employee’s performance belongs to a specific category (e.g., Good).

*Non-membership degree* $\nu _i$: If an employee’s performance score is far from the threshold for a given category, the non-membership degree $\nu _i$ will be high. Mathematically, it can be modeled as a linear function based on the difference from the performance threshold:

7 $$\begin{aligned} \begin{aligned} \nu _i&= 1 - \mu _i \\&\quad \text {(if the employee is unlikely to belong to a category)} \end{aligned} \end{aligned}$$

*Hesitation degree* $\pi _i$: The hesitation degree represents the ambiguity or uncertainty in deciding the membership. It is often calculated using:

8 $$\begin{aligned} \pi _i = 1 - (\mu _i + \nu _i) \end{aligned}$$

If the performance score is near the threshold for a category, the hesitation degree $\pi _i$ will be high, indicating uncertainty in classification. The employee is placed into one of the pre-defined performance categories of Good, Average, or Poor using computed membership, non-membership, and hesitation degrees. The categories are determined by the calculated fuzzy score $p_i^{\text {fuzzy}}$ and the fuzzy logic thresholds. For instance:

- If $\mu _i$ is greater than a predefined threshold $\mu _{\text {threshold}}$, the employee is classified as Good.
- If $\nu _i$ is greater than a predefined threshold $\nu _{\text {threshold}}$, the employee is classified as Poor.
- If neither condition is met, the employee is classified as Average.

#### Example: employee performance evaluation

Let’s consider an example where we have an employee with the following metrics:

- Previous year rating $p_1 = 4.5$,
- Average training score $p_2 = 3.8$,
- KPIs met more than 80% $p_3 = 0.9$.

The weights for these metrics are:

- $w_1 = 0.3$ for previous year rating,
- $w_2 = 0.25$ for average training score,
- $w_3 = 0.45$ for KPIs met.

The weighted performance score $p_i^{\text {fuzzy}}$ is calculated as:

9 $$\begin{aligned} p_i^{\text {fuzzy}}&= 0.3 \times 4.5 + 0.25 \times 3.8 + 0.45 \times 0.9 \nonumber \\&= 1.35 + 0.95 + 0.405 = 2.705 \end{aligned}$$

The sigmoid function gives the membership degree:

$$\begin{aligned} \mu _i = \frac{1}{1 + e^{-2.705}} \approx 0.937 \end{aligned}$$

Assuming the non-membership degree $\nu _i$ and hesitation degree $\pi _i$ are computed as:

$$\begin{aligned} \nu _i = 1 - \mu _i \approx 1 - 0.937 = 0.063 \\ \pi _i = 1 - (\mu _i + \nu _i) = 1 - (0.937 + 0.063) = 0 \end{aligned}$$

This employee would be classified as Good based on a high membership degree and low non-membership.

### Deep learning enhancement

**Fig. 2:**
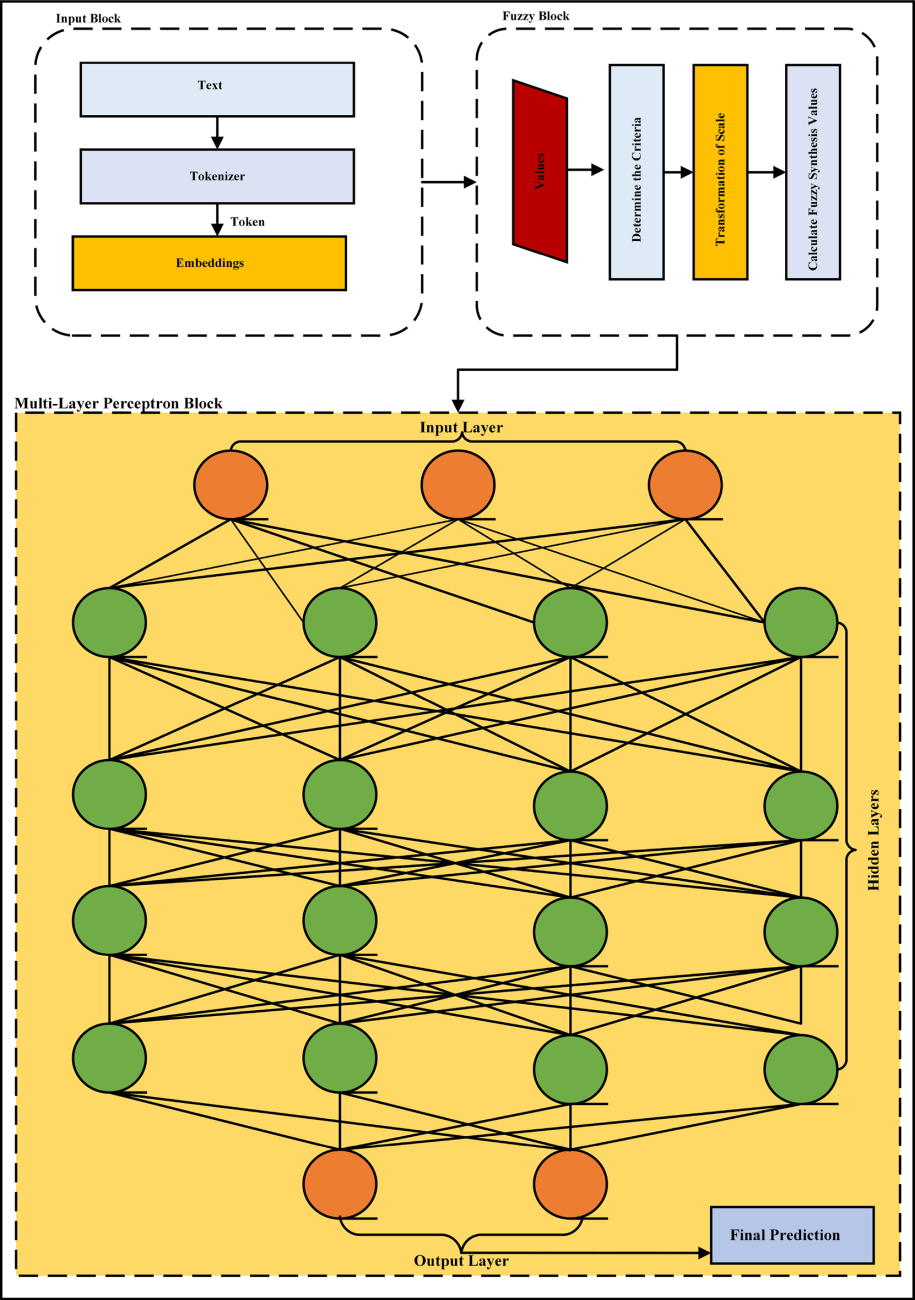
Diagram of the proposed model architecture, consisting of an input block for text processing, a fuzzy block for handling uncertain data, and a multi-layer perceptron block for generating final predictions.

To improve the accuracy of the employee performance predictions, Deep Learning (specifically, multi-layer perceptron) is used as shown in Fig. 2, to further refine the fuzzy performance scores as predicted earlier.

Let $p_i^{\text {fuzzy}}$ be the fuzzy performance score for employee *i*, and $x_i^{\text {fuzzy}}$ represent the input features for the deep learning model (i.e., fuzzy evaluation scores).

- *Neural network architecture* The neural network consists of three fully connected layers with $n_1 = 128$, $n_2 = 64$, and $n_3 = 32$ neurons. The output of the neural network at each layer is computed as:

10 $$\begin{aligned} a^{(l)} = \sigma \left( W^{[l]} a^{[l-1]} + b^{[l]}\right) \end{aligned}$$

where:

- $a^{(l)}$ is the activation of the *l*-th layer,
- $W^{[l]}$ and $b^{[l]}$ are the weight matrix and bias vector for the *l*-th layer,
- $\sigma (\cdot )$ is the activation function (e.g., ReLU or Sigmoid),
- $a^{[l-1]}$ is the input to the *l*-th layer (with $a^{[0]} = x_i^{\text {fuzzy}}$).

The neural network is trained using the Mean Squared Error (MSE) loss function, given by:

11 $$\begin{aligned} \text {MSE} = \frac{1}{N} \sum _{i=1}^{N} \left( y_i^{\text {pred}} - p_i^{\text {fuzzy}} \right) ^2 \end{aligned}$$

where $y_i^{\text {pred}}$ is the predicted performance score, and $p_i^{\text {fuzzy}}$ is the fuzzy score. The network parameters *W* and *b* are updated using the Adam optimizer to minimize the loss function.

#### Resource allocation optimization

Once the employee performance scores are computed using both fuzzy logic and deep learning, the next step is to allocate resources (training hours and mentorship sessions) efficiently.

Let $R = \{r_{\text {train}}, r_{\text {mentor}}\}$ represent the resources to be allocated, where:

- $r_{\text {train}}$ is the number of training hours,
- $r_{\text {mentor}}$ is the number of mentorship sessions allocated to each employee.

**Training Hours Allocation:** The number of training hours allocated to employee *i* is a function of the employee’s predicted performance score $y_i^{\text {pred}}$ and their performance category:

$$\begin{aligned} r_{\text {train},i} = f_{\text {train}}(y_i^{\text {pred}}, \text {category}) \end{aligned}$$

where $f_{\text {train}}$ is a rule-based heuristic function which allocates more resources to high-priority employees. The $f_{\text {train}}$ function allocates training hours by category: critical employees receive 40 hours, high-priority 30 hours, medium-priority 20 hours, and low-priority 10 hours, totaling 537,560 hours for 17,417 employees.

**Mentorship Sessions Allocation:** Similarly, the number of mentorship sessions is allocated based on the predicted performance score:

$$\begin{aligned} r_{\text {mentor},i} = f_{\text {mentor}}(y_i^{\text {pred}}, \text {category}) \end{aligned}$$

The $f_{\text {mentor}}$ is a rule-based heuristic function which assigns mentorship sessions as 20% of training hours, resulting in 107,512 sessions. These transparent, deterministic functions convert performance assessments into resource allocation recommendations that scale with employee count and performance distribution.

**Total Cost:** The total cost $C_{\text {total}}$ of the resource allocation is the sum of the costs associated with training hours and mentorship sessions:

12 $$\begin{aligned} C_{\text {total}} = \sum _{i=1}^{N} \left( r_{\text {train},i} \cdot \text {Cost per Hour} + r_{\text {mentor},i} \cdot \text {Cost per Session} \right) \end{aligned}$$

where the *Cost per Hour* and *Cost per Session* are constants representing the cost associated with each resource.

## Experimental results and evaluation

In this section, we present the findings of the experimental procedure to demonstrate the proposed framework which integrates the use of IFZN with a Multi-layer perceptron for employee performance evaluation and resource allocation. The evaluation will consist of the accuracy with which performance can be predicted, the relationship to which the fuzzy result aligns with deep learning, and the strategies for resource allocation.

The HR Analytics dataset comprises 17,417 employee records, encompassing 13 original attributes that provide extensive organizational and performance data. This dataset includes unique employee identification numbers, an organizational structure featuring 9 departments (Technology, HR, Sales & Marketing, Procurement, Finance, Analytics, Operations, Legal, R&D), and 34 geographic regions. It also contains demographic information, such as education levels (Below Secondary, Bachelors, Masters & above) with 771 missing values, gender distribution, and employment details, including recruitment channels, age range (21-58 years), and service length (1-26 years). Performance metrics are represented by previous year ratings (1-5 scale) with 1,363 missing values, average training scores (39-99 range), number of training sessions (1-10), and achievement indicators, including KPI achievement (binary) and awards won (binary). The dataset demonstrates a data quality of 95.2%, with 2,134 missing cells out of a total of 226,421, primarily affecting education (4.4% missing) and previous year ratings (7.8% missing). Following preprocessing, the dataset expands to 23 columns through the normalization of 5 numeric features and the encoding of 5 categorical features, serving as the basis for implementing Intuitionistic Fuzzy Z-Numbers and Deep Learning methodologies for employee performance evaluation and resource allocation optimization across diverse organizational contexts.

**R**$^{2}$ **Score:** The $R^2$ score measures the proportion of variance in the dependent variable that is predictable from the independent variables. A higher $R^2$ indicates better model fit.

13 $$\begin{aligned} R^2 = 1 - \frac{\sum _{i=1}^{N}(y_i^{\text {pred}} - y_i)^2}{\sum _{i=1}^{N}(y_i - {\bar{y}})^2} \end{aligned}$$

where:

- $y_i^{\text {pred}}$ is the predicted performance score for employee *i*,
- $y_i$ is the actual performance score,
- ${\bar{y}}$ is the mean of the actual scores.

**MSE:** The MSE evaluates the average squared difference between the predicted and actual values. The formula for MSE is:

14 $$\begin{aligned} \text {MSE} = \frac{1}{N} \sum _{i=1}^{N} (y_i^{\text {pred}} - y_i)^2 \end{aligned}$$

**Correlation Coefficient:** The correlation coefficient is used to assess the strength of the relationship between the fuzzy and deep learning predictions. The formula is:

15 $$\begin{aligned} \text {Correlation} = \frac{\sum _{i=1}^{N}(y_i^{\text {fuzzy}} - {\bar{y}}_{\text {fuzzy}})(y_i^{\text {pred}} - {\bar{y}}_{\text {pred}})}{\sqrt{\sum _{i=1}^{N}(y_i^{\text {fuzzy}} - {\bar{y}}_{\text {fuzzy}})^2} \sqrt{\sum _{i=1}^{N}(y_i^{\text {pred}} - {\bar{y}}_{\text {pred}})^2}} \end{aligned}$$

where $y_i^{\text {fuzzy}}$ and $y_i^{\text {pred}}$ are the fuzzy and deep learning predictions, respectively, and ${\bar{y}}_{\text {fuzzy}}$ and ${\bar{y}}_{\text {pred}}$ are their respective means.

The framework provides a complete suite of results and visualizations. The experimental process produced a number of insights into employee performance and contributions across departments, uncertainty in performance feedback, and findings on environmental resource needs. The data from the framework was aggregated into 21 standalone visualizations that were developed through the analysis process.

**Fig. 3:**
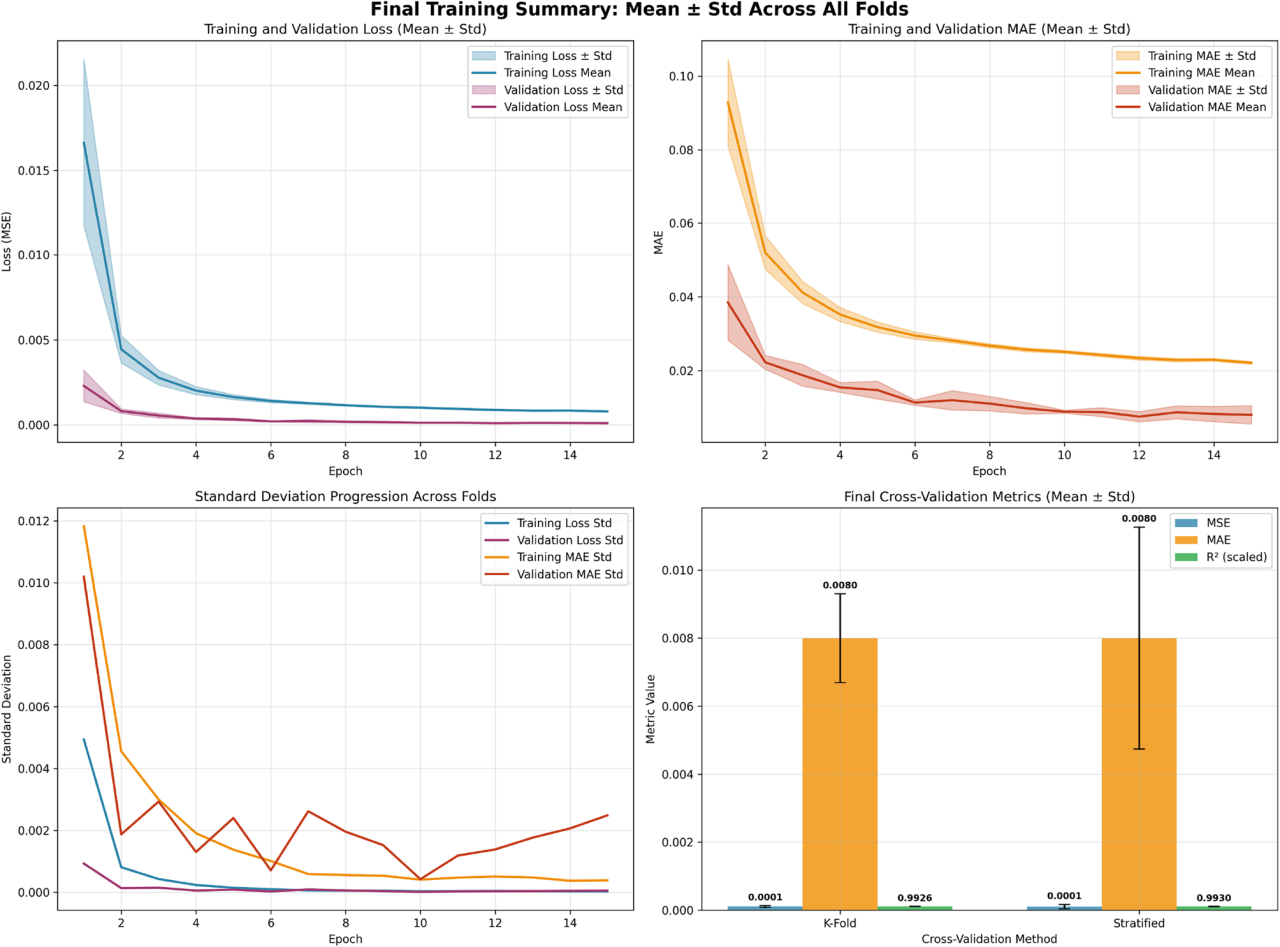
Final training summary and cross-validation results.

**Training Setup:** The multilayer perceptron (MLP) training runs for 30 epochs with early stopping to prevent overfitting. Cross-validation uses 20 epochs per fold. The final model training uses 30 epochs, with early stopping patience of 10 epochs. Dropout layers (0.3, 0.2, 0.1) follow each dense layer, while batch normalization follows the first two dense layers to stabilize training. Early stopping with restore_best_weights=True halts training when validation loss stabilizes. The MLP uses a three-layer architecture (128$\rightarrow$64$\rightarrow$32 neurons) with dropout regularization and batch normalization. Training employs Adam optimizer with MSE loss, batch size of 32, and early stopping. The model processes 17,417 employee records with 17 features, achieving an R$^{2}$ of 0.6330 in the ablation study. The training process is shown in Fig. 3, presenting a comprehensive summary of the model’s performance throughout the training process and its evaluation across different cross-validation techniques. The top-left plot shows the training and validation loss (MSE), with both losses rapidly decreasing in the early epochs and stabilizing, indicating the model is effectively learning and generalizing to the validation data. The validation loss slightly trails behind the training loss, exhibiting a minimal variance as training progresses. Similarly, the top-right plot displays the training and validation MAE, where both metrics decrease over time, with the validation MAE remaining slightly lower, suggesting the model’s effective generalization despite slight variance. The bottom-left plot visualizes the standard deviation of both the training loss and MAE across epochs, showing a reduction in variance, which implies that the model’s predictions become more stable and consistent as training advances. Finally, the bottom-right plot compares the final cross-validation metrics for K-Fold and Stratified methods, showing both techniques yield low MSE values and high R$^{2}$ scores, with the Stratified method slightly outperforming K-Fold in R$^{2}$ but both methods demonstrating strong predictive accuracy. Overall, the figure underscores the model’s effectiveness, stability, and reliability in both training and cross-validation.

### Employees performance evaluation and visualization insights

**Fig. 4:**
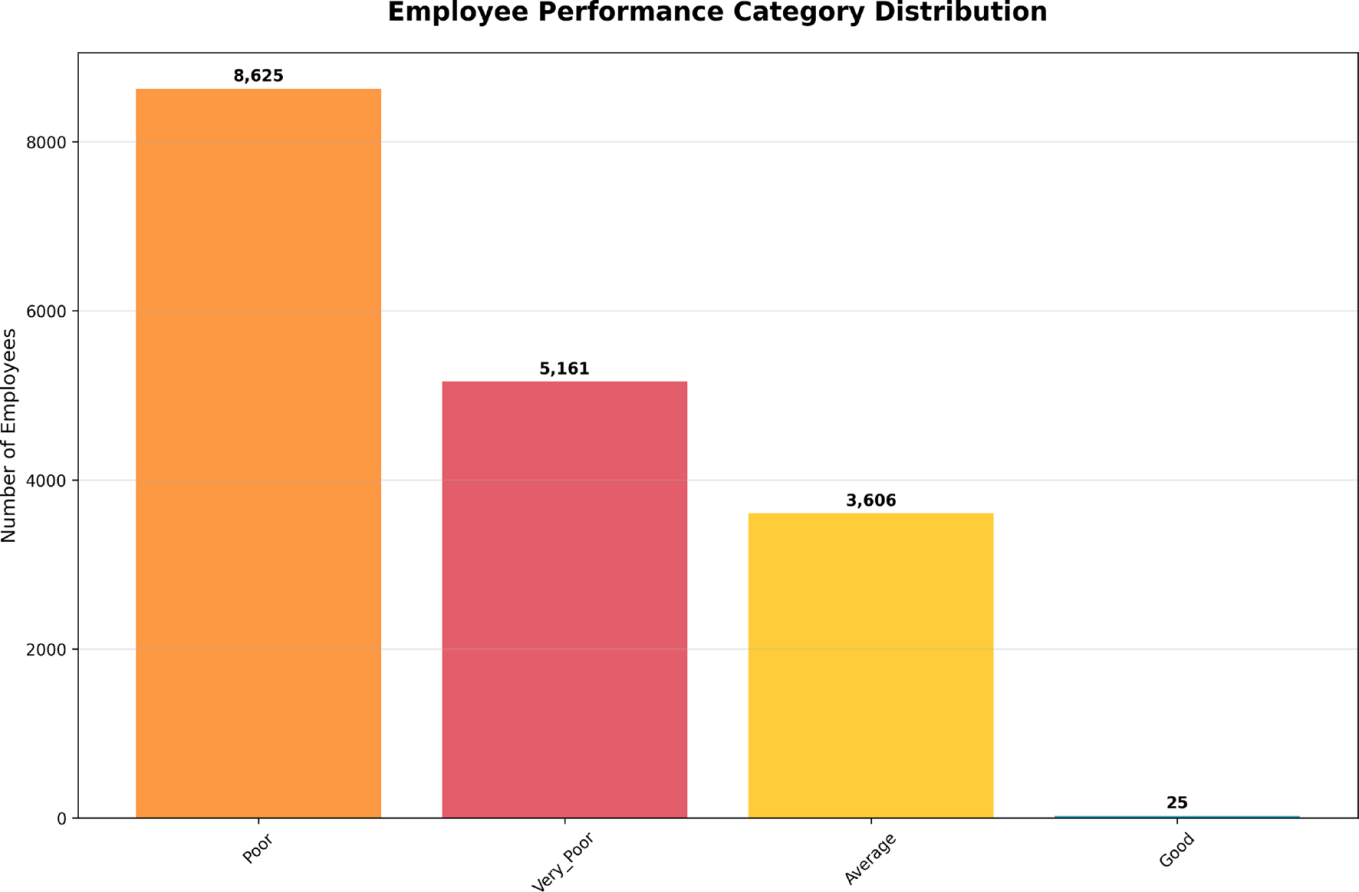
Distribution of employees across performance categories, with the majority in the Poor and Very Poor categories.

The Performance Distribution in Fig. 4 clearly demonstrates the distribution of employees in three performance categories: Good, Average, Poor, and Very Poor. According to the chart, the largest group of employees is Poor, 8625 in total. Next, Very Poor with 5161 employees. The average employee is 3606, while only 25 employees are rated as Good, which is due to the high criteria set for performance evaluation.

This distribution indicates several employees are falling short of expectations, as the majority of employees are Poor and Very Poor. Based on these results, it indicates immediate action is needed, including additional training and mentoring programs to further improve these employees’ performance. Perhaps the Good performance group, although small, could serve as the ideal workforce group to foster and encourage further desired performance growth.

As Fig. 4 data highlights, organizations need to allocate resources effectively in improvement programs, particularly focusing on the lower-performing groups.

**Fig. 5:**
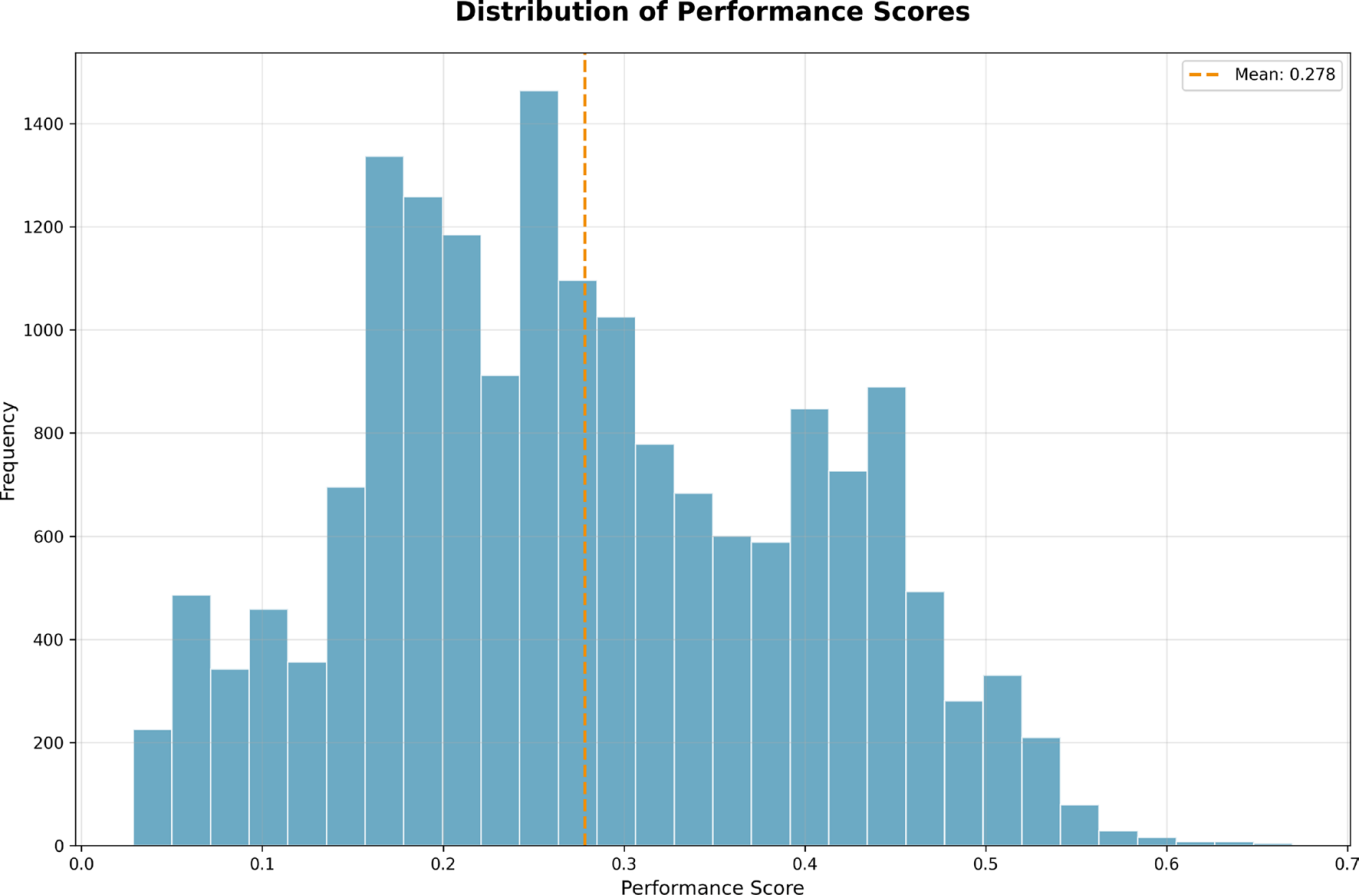
Distribution of employee performance scores, with the mean score marked at 0.278, highlighting a concentration of employees in the lower performance ranges.

The Performance Histogram as shown in Fig. 5, displays the distribution of employee performance scores ranging from 0.0 to 0.7. The majority of employees score within the range of 0.20 to 0.30, meaning that the vast majority of employees are under-performing in their jobs.

The histogram indicates an average performance score of 0.278, which is represented in these histograms showing the upper and lower performance ranges. The histogram has a distributed skew with more employees falling into the lower performance ranges (0.1 to 0.3). A greater number of employees are also engaged in lower performance ranges and indicates that all lower performing employees need targeted training and mentorship.

There are fewer employees in higher performance levels (0.4 to 0.6), with those employees engaged in a higher performance level indicating that top performers are few and far between. This sort of distribution further implies the need for targeted training to reduce the lower employee proportion in the histogram and raise overall employee performance levels, in addition to investing in top performers to prepare them for leadership roles.

**Fig. 6:**
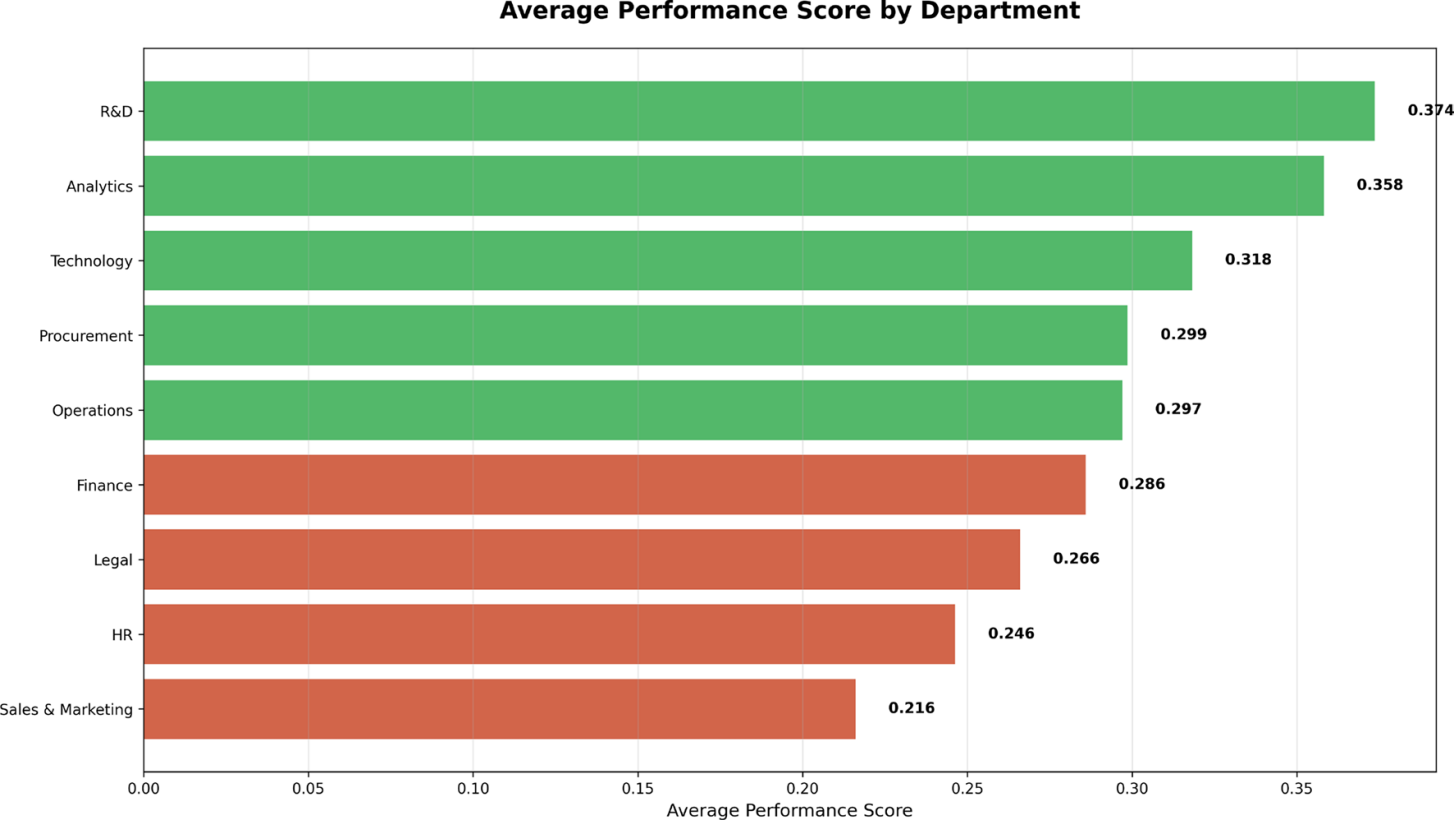
Department-wise average performance scores. Green bars indicate higher-performing departments, while red bars highlight departments requiring improvement.

The Department Performance in Fig. 6 depicts average performance across different departments. R&D and Analytics had the best performance, with scores of 0.374 and 0.358, respectively. Sales & Marketing, HR, and Legal had much lower scores, with HR showing the lowest performance at 0.216.

The green bars indicate departments with good performance, while red bars show areas needing improvement. This graphic illustrates where the organization performs well and where it must target interventions (such as training or process modifications) to raise performance in the poorer-performing departments.

**Fig. 7:**
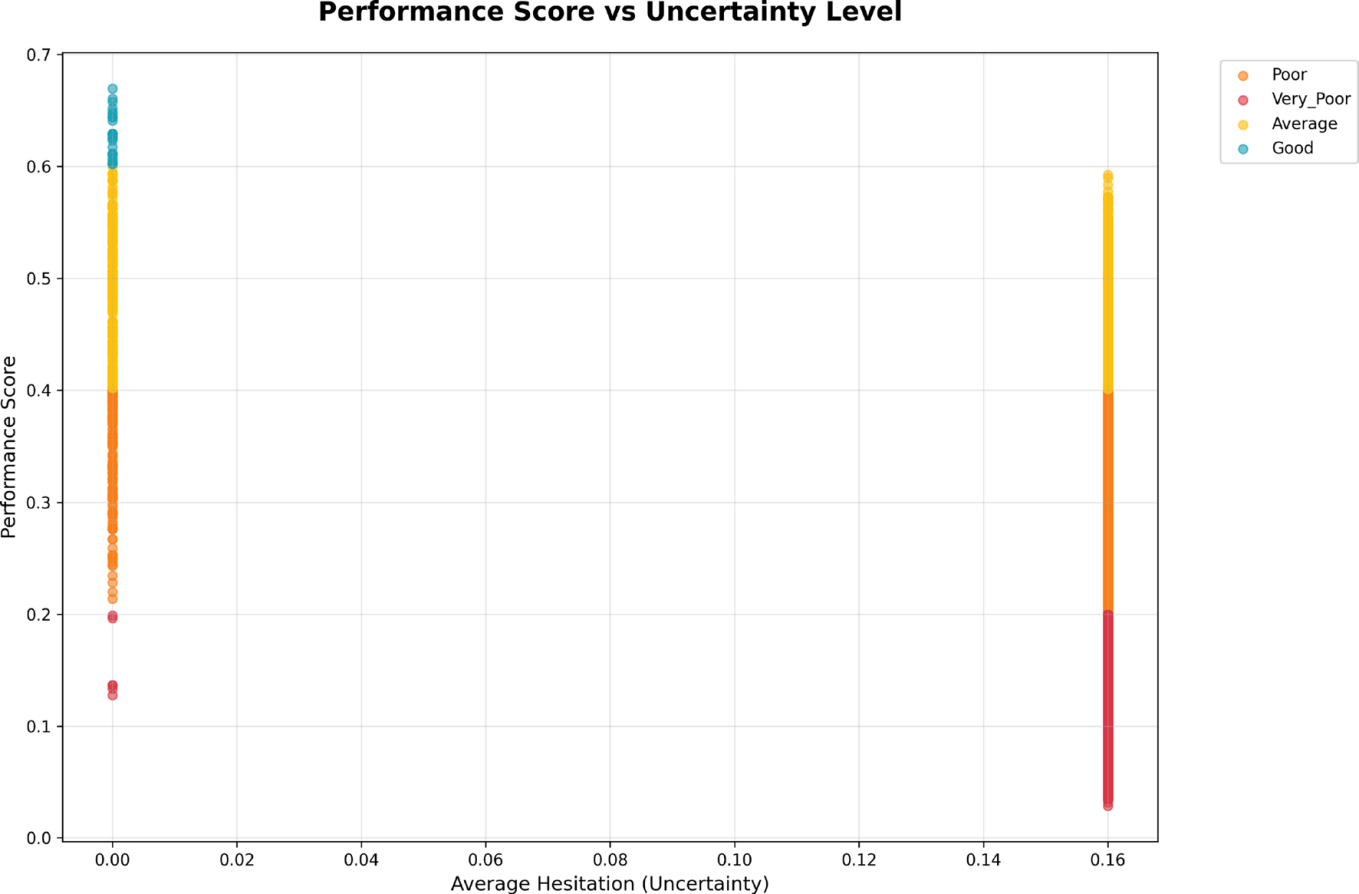
Scatter plot showing the relationship between employee performance scores and the hesitation level.

The Performance vs. Uncertainty scatter plot shown in Fig. 7 describes the relationship between employee performance scores and the associated uncertainty in their evaluations. The *x*-axis represents the average hesitation (uncertainty) level, conveying how uncertain the performance evaluation is for each employee. The *y*-axis shows the performance score, ranging from 0.0 to 0.7.

Each point is color-coded by performance category:

- **Red** - Poor
- **Orange** - Very Poor
- **Yellow** - Average
- **Cyan** - Good

When examining the plot in Fig. 7, it is clear that employees with high performance scores (close to 0.6 or 0.7) have a low level of uncertainty in their evaluations, indicating that confidence in their performance evaluation is high.

Whereas, employees in the Poor and Very Poor performance categories, on the other hand, exhibit a higher level of uncertainty, particularly those rated around 0.1 to 0.2 may face multiple inconsistencies when evaluated for performance. This variability suggests that perhaps an incomplete data set influenced the ratings or even subjectivity by the evaluator.

The performance evaluation implies that higher performing employees have a more confident assessment of their performance evaluation, whereas the lower performing employees exhibit significant uncertainty in the performance category.

**Fig. 8:**
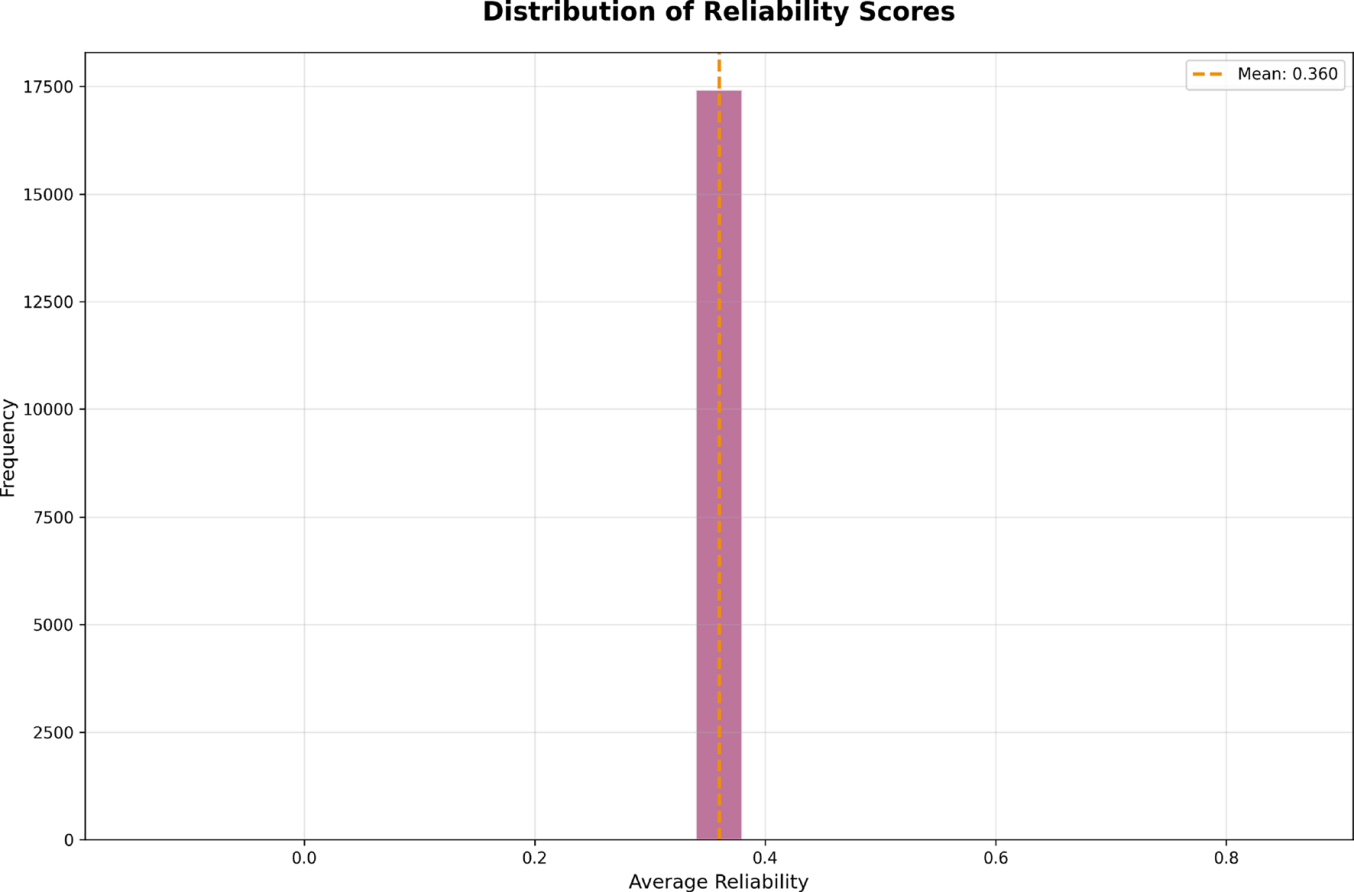
Distribution of reliability scores across employees, highlighting the concentration of evaluations with moderate uncertainty.

The Reliability of the employees in Fig. 8 displays the distribution of reliability scores across employees, providing information on the degree of certainty of the performance assessments. In the histogram, the x-axis represents the average reliability score, which runs from 0.0 to 0.8; the y-axis shows the frequency of employees in the various ranges of reliability scores.

The orange dashed vertical line at 0.360 represents the mean reliability score. This shows that most performance assessments for employees have a moderate degree of certainty. The histogram shows that most employees fall around this mean, suggesting that most performances are considered fairly reliable or have some confidence.

The histogram also has a slight tail toward the maximum end, which indicates even fewer employees with assessments that are more reliable or with less uncertainty.

The overall takeaway from the plot in Fig. 8 is that lower performing employees require better evaluation criteria or improved data collection processes to improve the credibility of their performance assessment in future assessments, so they need attention from authorities.

**Fig. 9:**
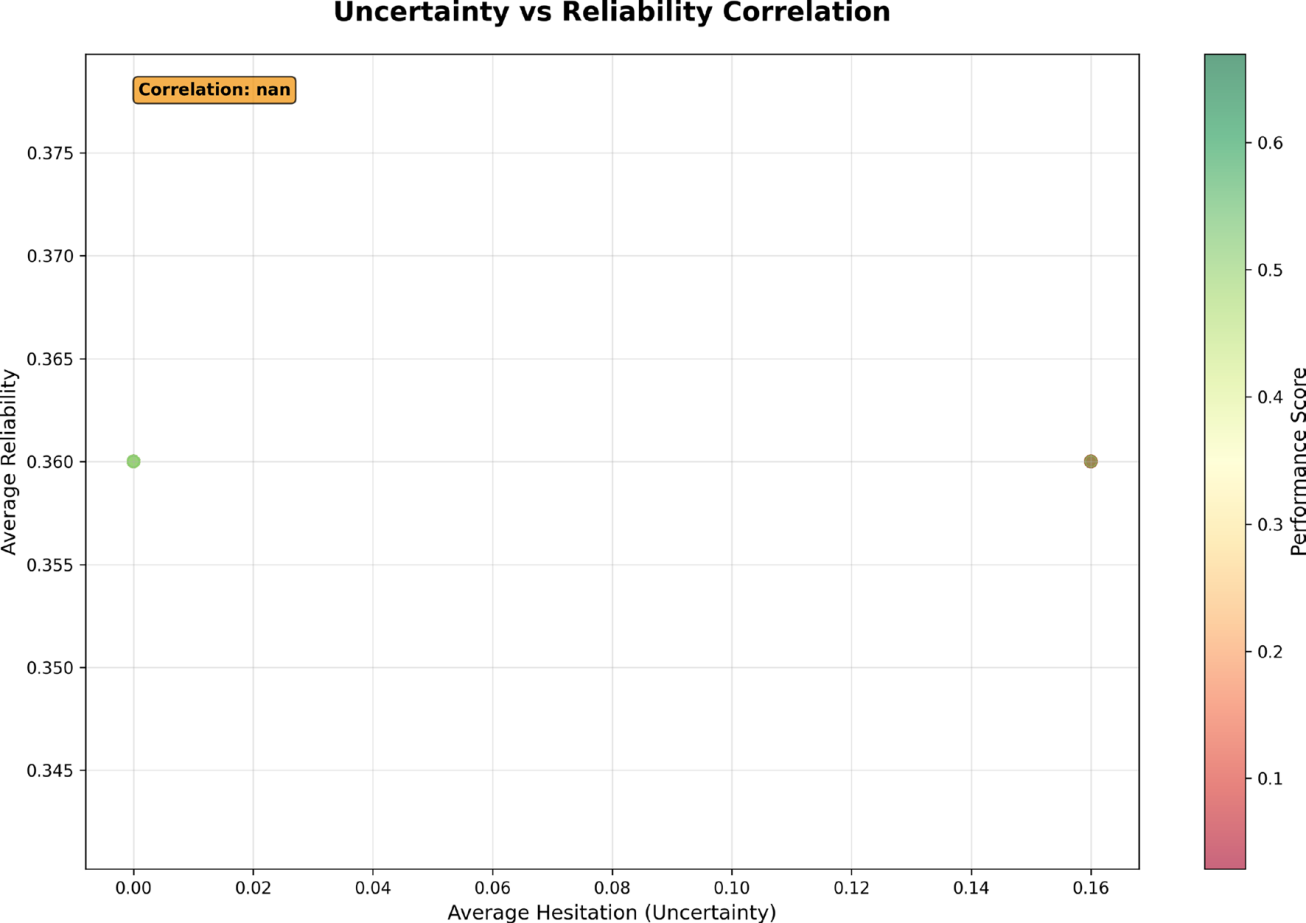
Uncertainty vs. Reliability Correlation, showing the relationship between uncertainty in performance evaluations and their reliability.

The Uncertainty vs. Reliability Correlation shown in Fig. 9 provides an illustration of the relationship between average hesitation (uncertainty) and the average reliability in employee performance evaluations. The *x*-axis represents the uncertainty in performance evaluations, where higher values indicate greater hesitation or uncertainty. The *y*-axis represents the reliability of evaluations, with higher values indicating more reliable and consistent evaluation results.

Each point plotted corresponds to a set of employees, and the color scale reflects their performance scores (green for high performers and red for lower performers). The plot shows a single data point with a correlation value labeled “Correlation: nan”, indicating no observable relationship between uncertainty and reliability in this dataset. In other words, uncertainty in performance evaluations does not appear to correlate with their reliability, suggesting that further investigation or a larger dataset may be required to draw meaningful conclusions.

**Fig. 10:**
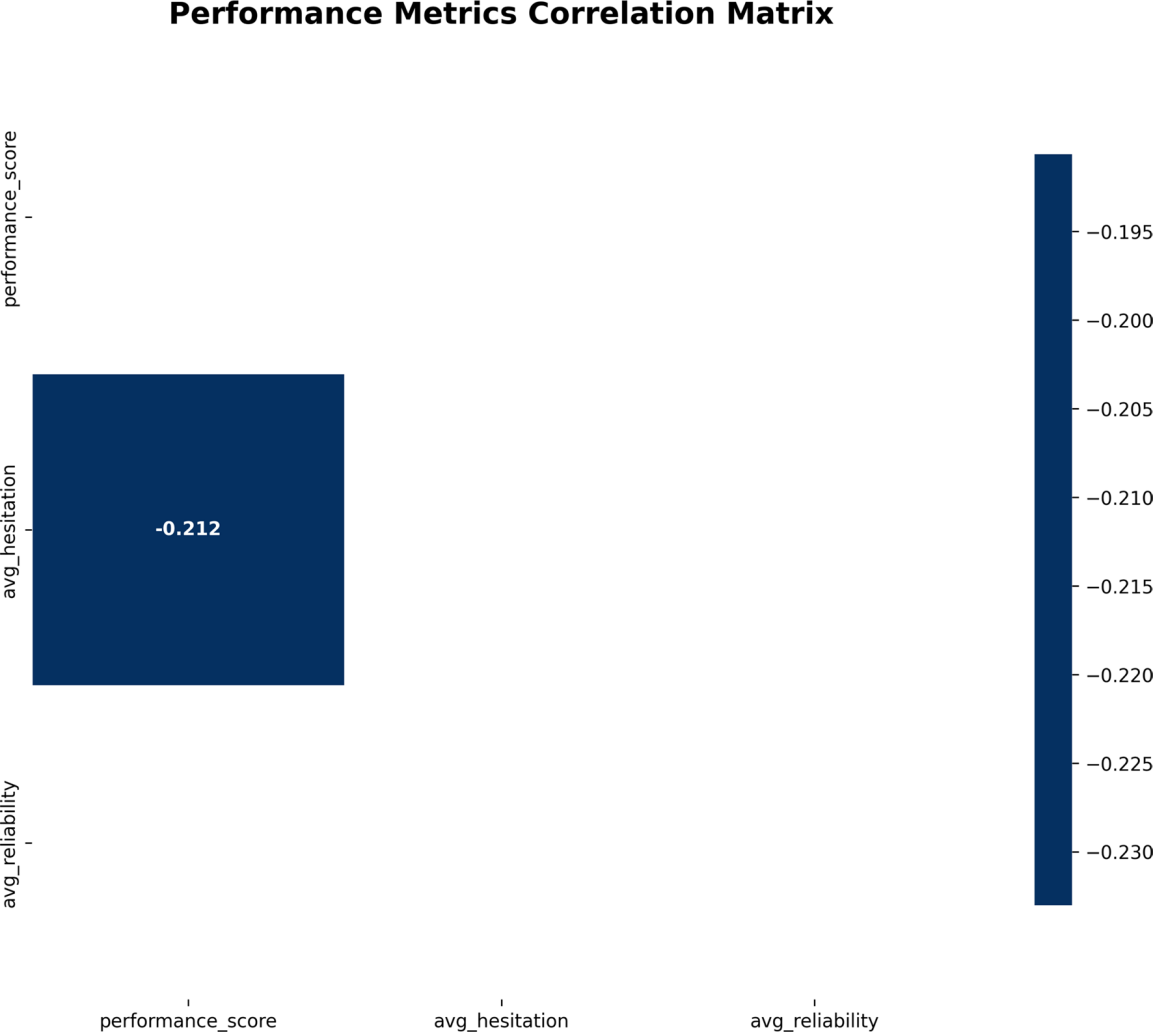
Performance metrics correlation matrix showing weak negative correlations between performance score, average hesitation (uncertainty), and average reliability.

The Performance Metrics Correlation Matrix in Fig. 10 depicts the correlations between the three main performance metrics: performance score, average hesitation (uncertainty), and average reliability. The correlation values are shown in the matrix and are visually represented through color intensity; darker intensity indicates a stronger correlation.

The chart shows a correlation of $-0.212$ between performance score and average hesitation, which is a weak negative correlation. This means that as uncertainty (hesitation) in the performance evaluation increases, the performance score tends to decrease slightly. We can also observe weak negative correlations between performance score and average reliability, as well as between average hesitation and average reliability.

Thus, there seems to be some relationship between performance uncertainty and reliability, suggesting that the higher the degree of uncertainty, the less reliable the performance evaluative delivery. However, these relationships are weak, indicating that there may be other influencing factors or hidden performance characteristics distorting the correlation.

**Fig. 11:**
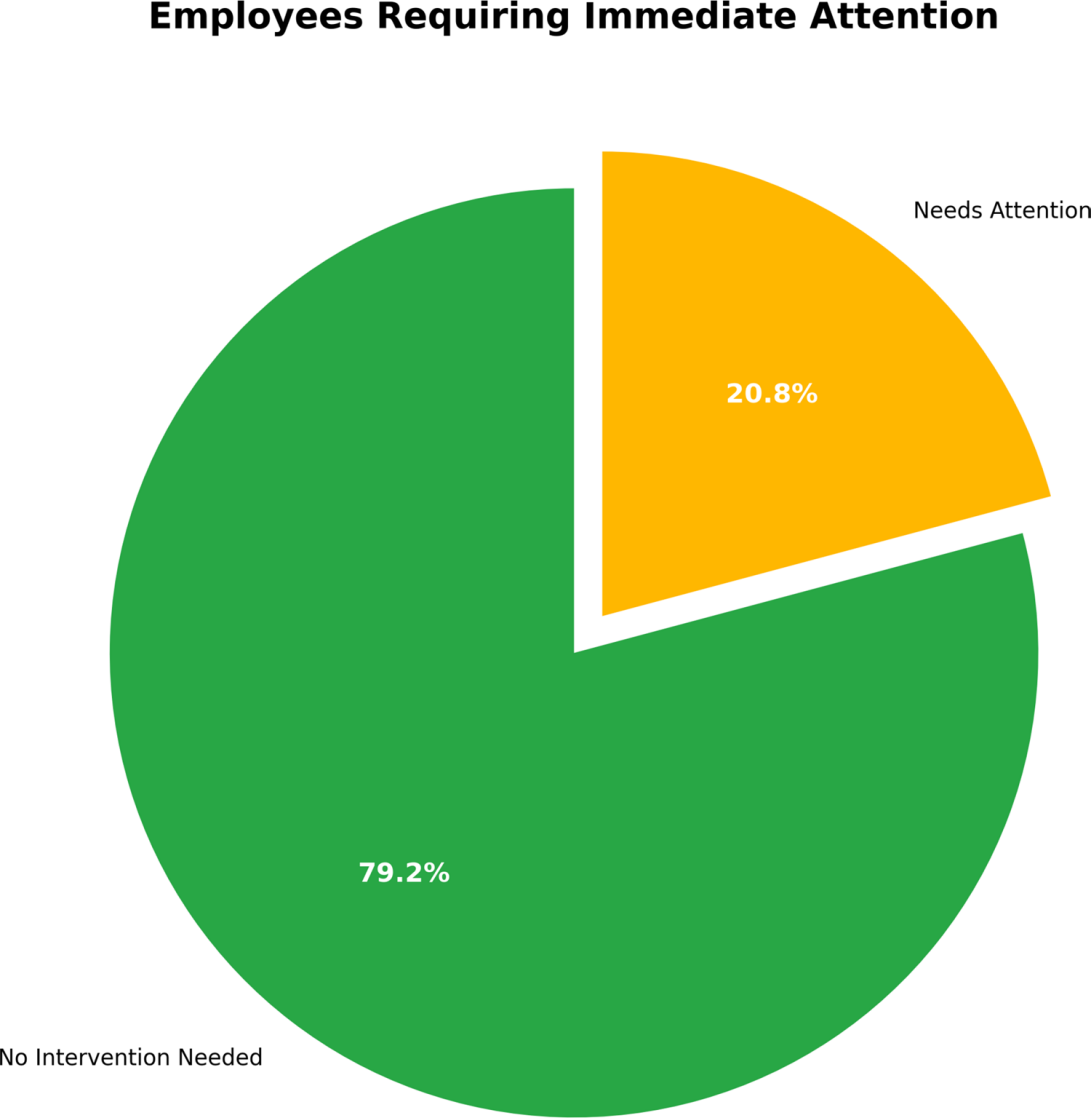
Distribution of employees requiring immediate attention.

The Needs Attention pie chart in Fig. 11 identifies the percentage of employees who underperformed based on the performance evaluation. As indicated in the chart, 20.8% of employees fall into the “Needs Attention” category (yellow). These individuals are apparently below the expected performance level and will require intervention (such as training, mentorship, or a performance improvement plan).

The remaining 79.2% of employees are listed in the “No Intervention Needed” category (green), because their performance levels were at acceptable levels and, at this time, did not require any action.

This visualization adds value for HR and management by identifying the proportion of employees who are underperforming and need additional resources or support to improve performance. At a minimum, it provides a clear avenue to distinguish who requires immediate attention. In this case, HR can focus on the 20.8% of employees who require intervention, while also indirectly supporting the success of the remaining workforce to improve overall organizational performance.

### Resource allocation insights

The experimental results also reveal key insights into the resource allocation process for training and mentorship based on employee performance. The resource allocation functions assign training hours based on performance categories: critical employees (5,161) are allocated 40 hours, high-priority employees (8,625) receive 30 hours, medium-priority employees (3,606) are given 20 hours, and low-priority employees (25) are assigned 10 hours, culminating in a total of 537,560 training hours. Mentorship sessions are allocated at 20% of the training hours, resulting in 107,512 sessions. The costs are calculated at $50 per hour for training and $100 per session for mentorship, leading to a total investment of $37,629,200. These functions employ weighted scoring derived from fuzzy logic results to establish priority levels and create department-specific resource distribution matrices.

**Fig. 12:**
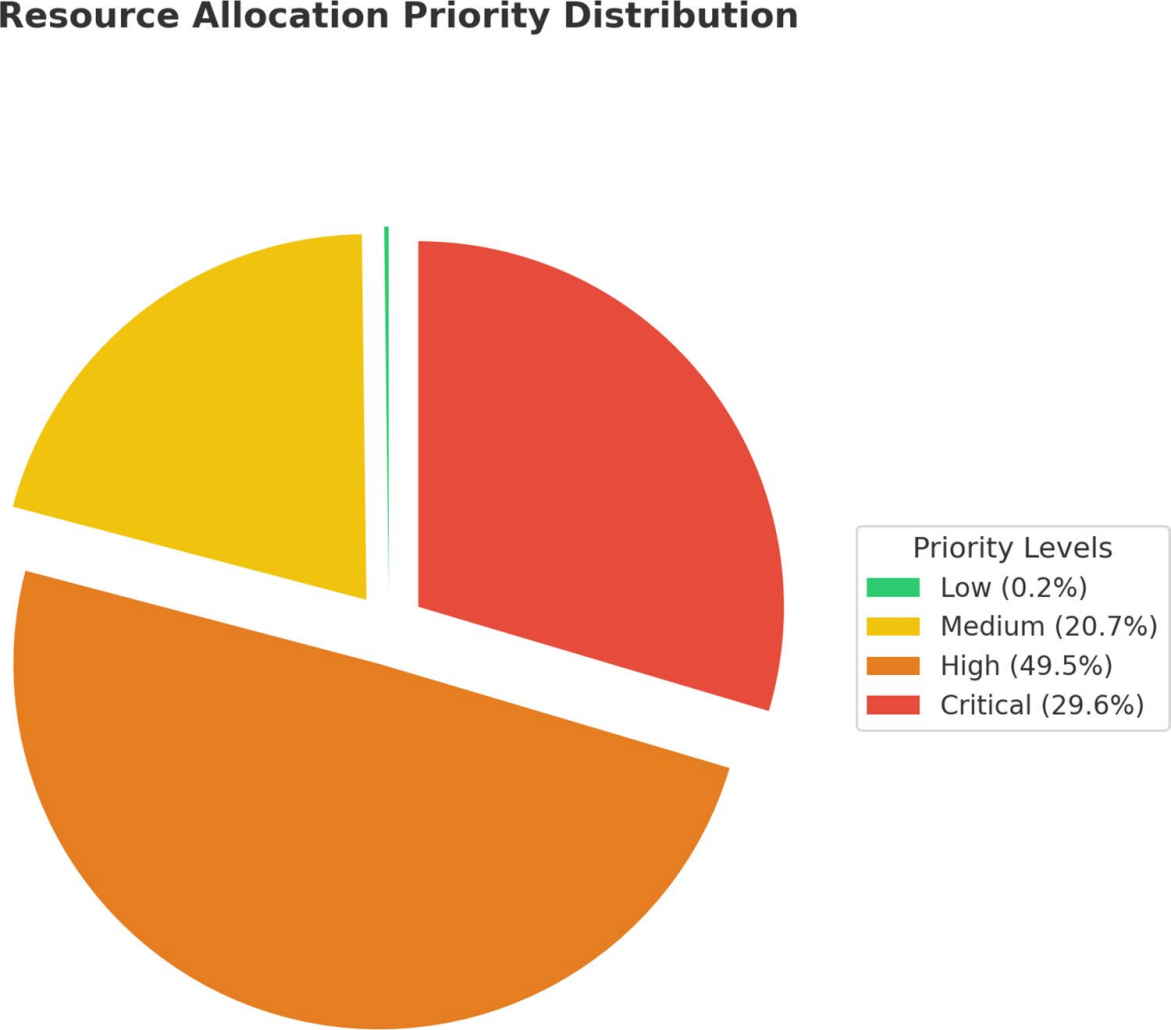
Distribution of resource allocation priorities.

The Resource Allocation Priority Distribution in Fig. 12 shows how resources (training hours, mentoring sessions, etc.) are allocated to employees based on performance evaluations by utilizing the priority rankings: Critical, High, Medium, and Low.

The High category (orange section), which includes 49.5% of employees, represents underperformers who need the largest allocation of resources among the four groups. The Critical group (red), consisting of 29.6% of employees, needs immediate support and intensive resource allocation. The Medium group (yellow section), covering 20.7% of employees, requires moderate intervention and resource deployment.

Finally, the Low group (green section), accounting for only 0.2% of employees, indicates there is no immediate intervention needed for a very small portion of the workforce. This distribution allows for a proper concentration of resources toward Critical and High priority employees, while providing intervention for the Medium group and negligible allocation for the Low group enabling more targeted employee development strategies.

**Fig. 13:**
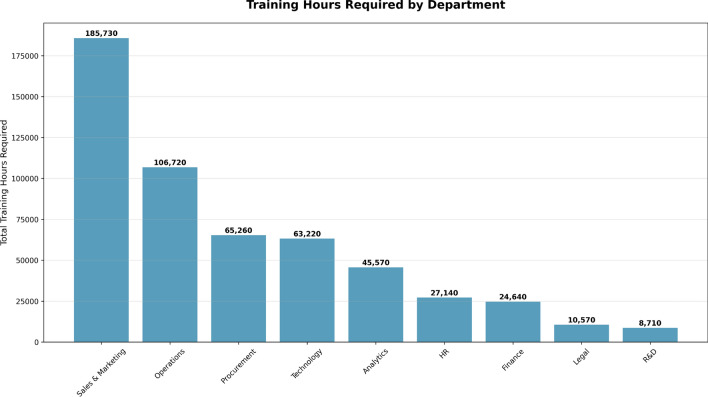
Total training hours required by each department, highlighting areas with the most significant need for training resources.

The Training Hours by Department chart, shown in Fig. 13, is closely related to the priority-based resource allocation. This visualization shows how training hours are distributed across departments based on performance needs.

Sales & Marketing is shown to require the most training hours (185,730), followed by Operations with 106,720 hours. This suggests these two departments have large performance gaps that demand immediate attention. Additionally, departments such as Technology (65,260 hours), Procurement (63,220 hours), and Analytics (45,570 hours) also require significant support to bridge skill gaps and enhance productivity.

By contrast, the hours required by departments with fewer training needs, such as HR, Finance, Legal, and R&D further indicate either a better level of performance or a lower priority for resource allocation. This chart enables HR departments to allocate training resources more effectively by prioritizing departments with the highest improvement potential. This allows organizations to capitalize on current opportunities and elevate overall workforce performance.

The training hours required by each department are also closely tied to the funding source allowable for those training activities. The total costs for training and mentorship are determined by the training hours used and the associated cost per unit of training or mentorship, incurred to support each department’s performance improvement efforts.

**Fig. 14:**
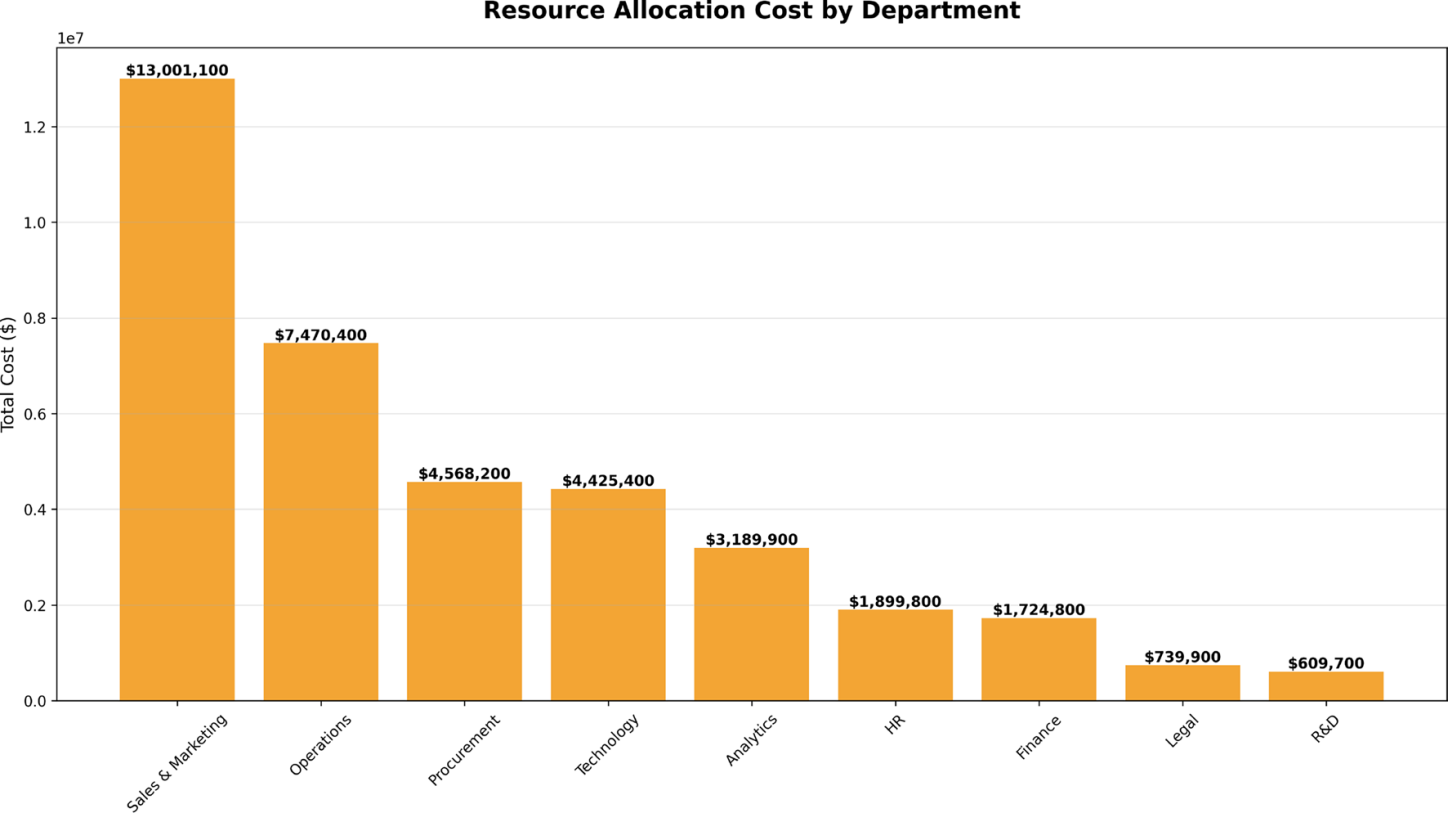
Total cost of training and mentorship resources required by each department to improve employee performance.

Figure 14 illustrates the total cost of training and mentorship resources allocated to each department. The chart provides insight into the financial investment tied to the training needs identified in the “Training Hours by Department” chart (Fig. 13), using two axes - with departments listed along the x-axis and total costs (in USD) on the y-axis.

The highest cost is associated with Sales & Marketing, which requires $13,001,100, reflecting the large number of training hours needed to close its performance gap. Other departments, such as Operations and Procurement, require $7,470,400 and $4,568,200, respectively. The lowest training costs are associated with the Legal and R&D departments, at $739,900 and $609,700, respectively.

This cost distribution summarizes where the largest investments are needed and provides a clear financial overview of what is required to support improvements in employee performance across departments.

**Fig. 15:**
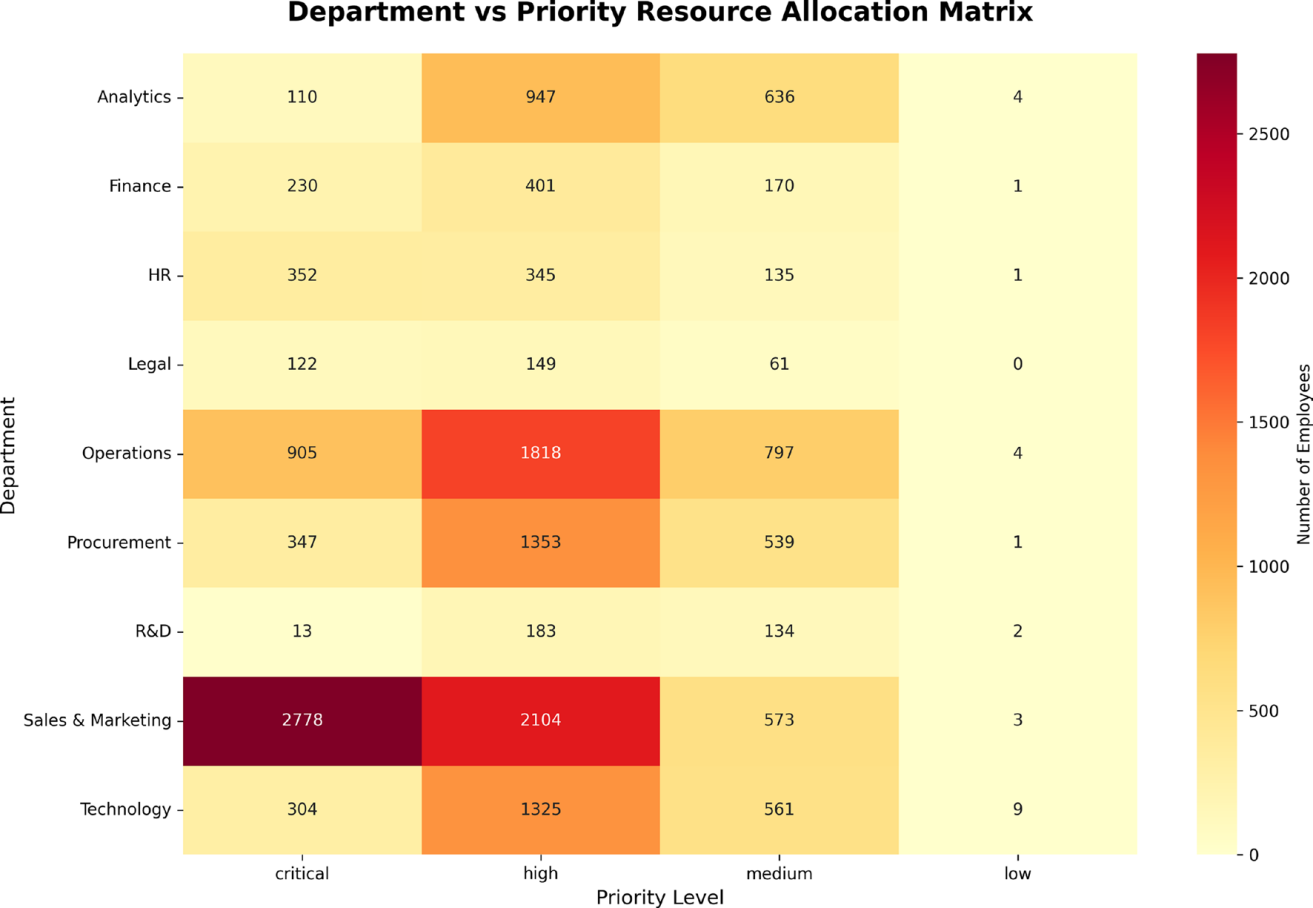
Resource allocation heatmap showing the distribution of employees across departments and priority levels, highlighting areas with the greatest resource needs.

The Resource Heatmap shown in Fig. 15 has a significant relationship with the Cost Analysis previously discussed. While the Cost Analysis provides a summary of the total financial commitment across all departments, Fig. 15 presents this same commitment at a more focused level, illustrating the distribution of resources amongst different priority levels within departments.

Understanding this distribution is important for decision-makers, as it reveals how training and mentorship resources are divided between priority groups such as *Critical*, *High*, *Medium*, and *Low*. This breakdown can help identify any inequities or imbalances in resource allocation, ensuring that support is fairly distributed based on actual need and performance deficits.

The Resource Heatmap depicts where resources (such as training hours and mentorship) are allocated across departments and priorities (Critical, High, Medium, and Low). The x-axis is priority, while the y-axis is the departments. The color intensity on the chart reflects the number of employees within each department and priority level, with darker colors representing more employees requiring more resources. It is most apparent in the heatmap that the Sales & Marketing department has the highest grouping of employees, with 2,778 employees in the Critical category and 2,104 in the High priority category. This is an area that would require more intervention through resources. The Operations department is also high priority with 1,818 employees in the High priority section which would require the same focused area of attention and intervention. Then we have areas like the Legal and R&D departments, which have less employees in the top two priority categories, the Legal department has no employees in the Critical category, which is a good sign meaning these areas require little intervention through resources.

The heatmap provides a relevant depiction of where resources are targeted and how HR and management can be assured that employees deserving the most intervention through resources receive that support.

### Uncertainty analysis

Uncertainty is an important contributor in performance evaluations, and the framework provides multiple approaches for assessing this uncertainty.

**Fig. 16:**
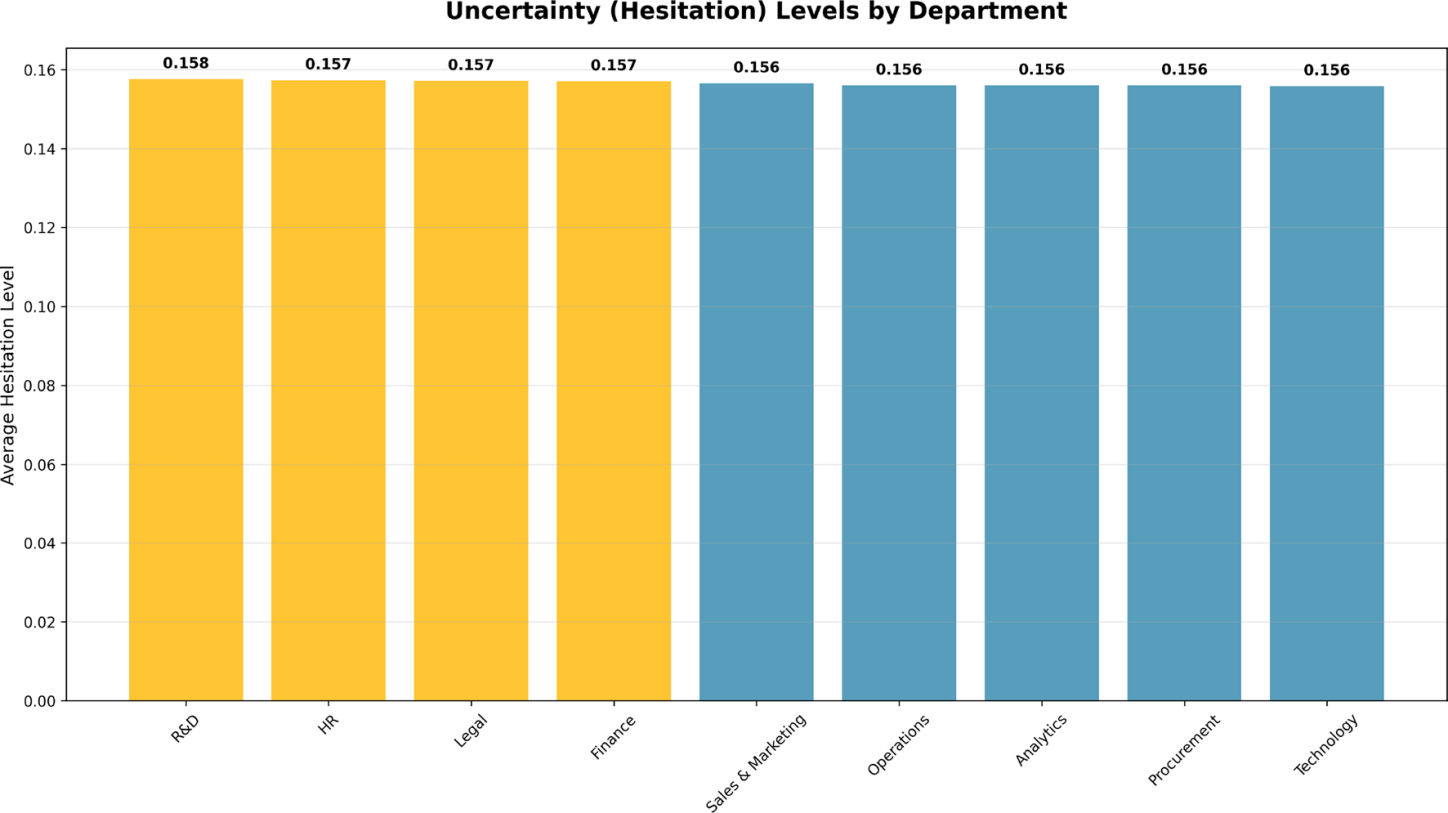
Average hesitation (uncertainty) levels across departments, highlighting areas with higher uncertainty in performance evaluations.

Figure 16 illustrates average hesitation (uncertainty) levels to the shaped the departments. The x-axis shows the departments while the y-axis shows the average hesitation level, vertically, exextendingtend from 0.0 to 0.16. The hesitation level shows how uncertain and the level of ambiguity of the performance evaluations are for employees in that department.

Based on the bar chart, departments that include R&D, HR, and Legal show slightly higher hesitation levels at about (0.157 and 0.158). Thus, these departments may have a little more uncertainty in their performance evaluations, indicating a possibility for improvement in the consistency and clarity of the performance evaluation metrics being used to evaluate performance. Conversely, departments like Sales & Marketing, Operations, and Technology also show relatively lower hesitation levels (0.156), which indicates that performance evaluations in these departments could be more trusted to be reliable and less uncertain in their results.

**Fig. 17:**
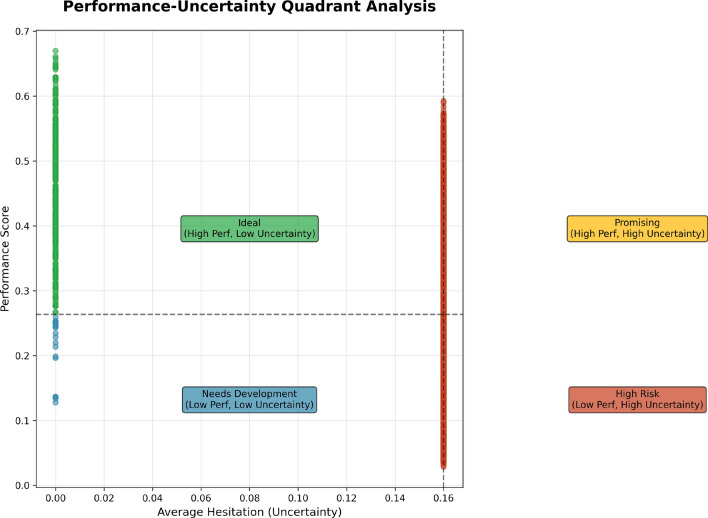
Performance-Uncertainty Quadrant Analysis categorizing employees into four quadrants based on performance and uncertainty levels, highlighting areas requiring focused intervention.

The overall estimates provided by the Hesitation bar chart guide areas where performance evaluations may need to be improved by clarifying performance evaluation criteria or collecting additional data that might provide greater clarification or reduce uncertainty. Of course, providing more accurate and reliable performance evaluations is the goal. The Performance-Uncertainty Quadrant Analysis Fig. 17 provides a unique employee classification scheme by presenting employees as plots by confidence and performance levels on the x and y-axis, respectively. The analysis divides the data among four quadrants in separate directions, each representing a distinct combination of performance and uncertainty levels. This analysis provides an opportunity to explore the four combinations for performance and uncertainty levels, which allows one to identify where employees perform at high uncertainty or low performance or any other scenario, to determine appropriate targeted strategies for improvement the areas of performance or confidence evaluation.

The Ideal Quadrant (green) represents employees with high performance and low uncertainty. These employees are doing great work and we are confident in their ratings, we do not need to do anything to assess their performance. The Promising Quadrant (yellow) includes employees with high performance but high uncertainty. These employees are performing well, but we are hesitant in our ratings, and need more clarity or data that we could use to evaluate their performance with solid clarity. The Needs Development Quadrant (blue) has employees with low performance/low uncertainty, it is obvious to them and to us clear areas to focus improvement efforts. The High-Risk Quadrant (red) represents employees with low performance and high uncertainty -those at the highest risk because they need the performance and evaluation to be addressed; we need to act urgently. This quadrant view of performance allows HR or management to act; any performance with uncertainty is a priority for intervention.

**Fig. 18:**
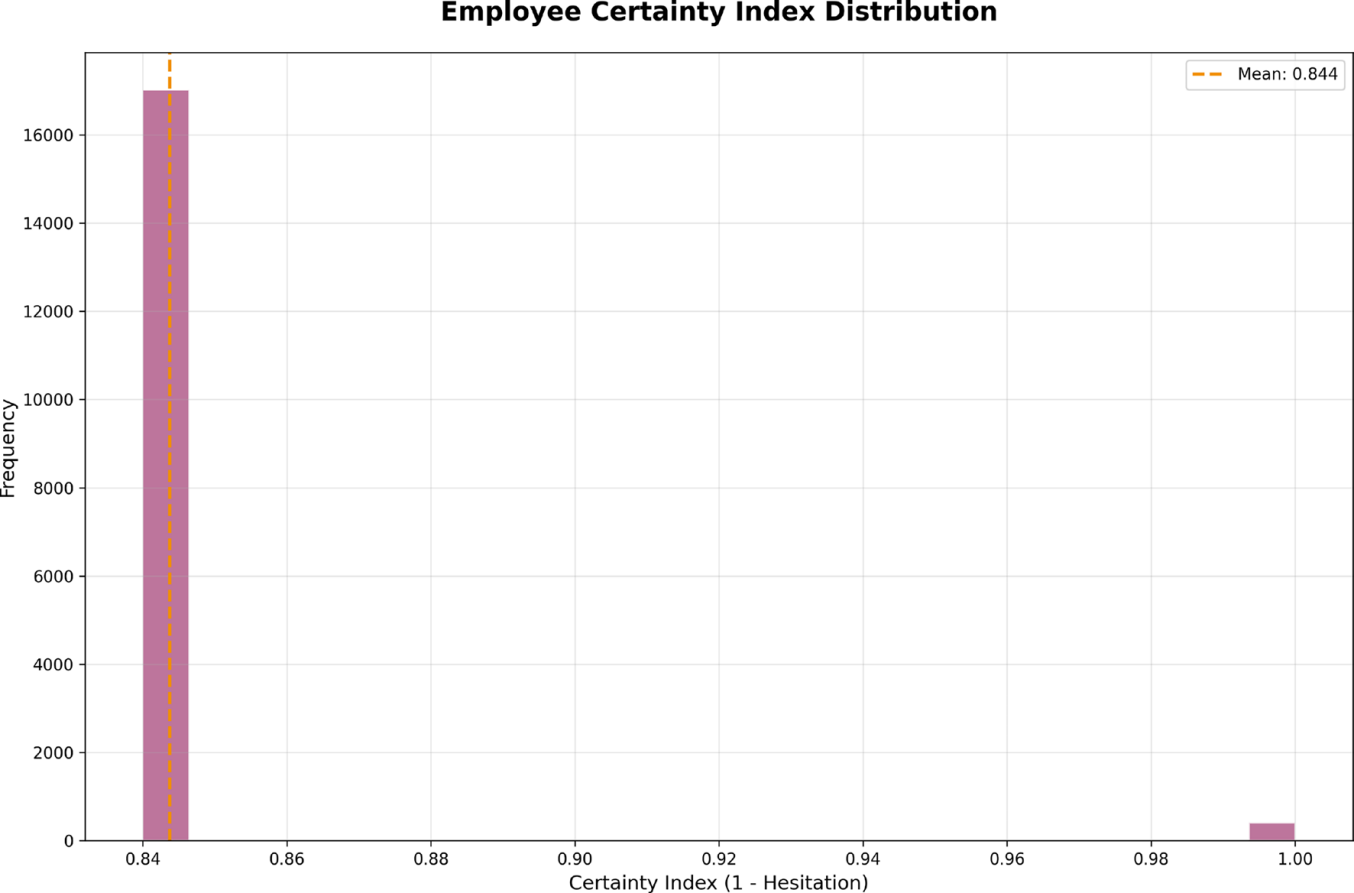
Distribution of the employee certainty index, showing the reliability of performance evaluations with a mean certainty index of 0.844.

The Certainty Index is the next step after the Performance-Uncertainty Quadrant Analysis, which will give you a clearer insight into how reliable performance evaluations are across the wider workforce. The Certainty Index histogram (Fig. 18) exhibits the certainty scores for employee performance evaluations, ranging from 0 to 1. The x-axis is the certainty index, with greater values (closer to 1) indicating more certainty in the evaluation and more reliable performance score. The y-axis indicates the frequency of employees in a given certainty index.

We can see from the chart that most employees have a certainty index near 0.84, and this level had the highest frequency, which would indicate that while most employee evaluations are relatively certain, we still have some degree of hesitation, or uncertainty. The mean Certainty Index is marked at 0.844, so overall we can generally be certain that employee performance evaluations were very certain; however, we do still have some employees with low certainty scores. While the tail of the histogram indicates some certainty indexes approaching 1 and that few employees had an evaluation with certainty index this high, this would indicate that most employees were rated with some confidence, but there are still a few assessments with near-perfect certainty. Figure 17 illustrates the opportunity to continue to adjust our performance evaluation approaches, specifically focusing on the few employees, whose evaluation certainty levels are lower than the rest of the employees, with an eye towards possibly achieving more reliable evaluations for all employees.

### Advanced analytics (performance × uncertainty × reliability)

**Fig. 19:**
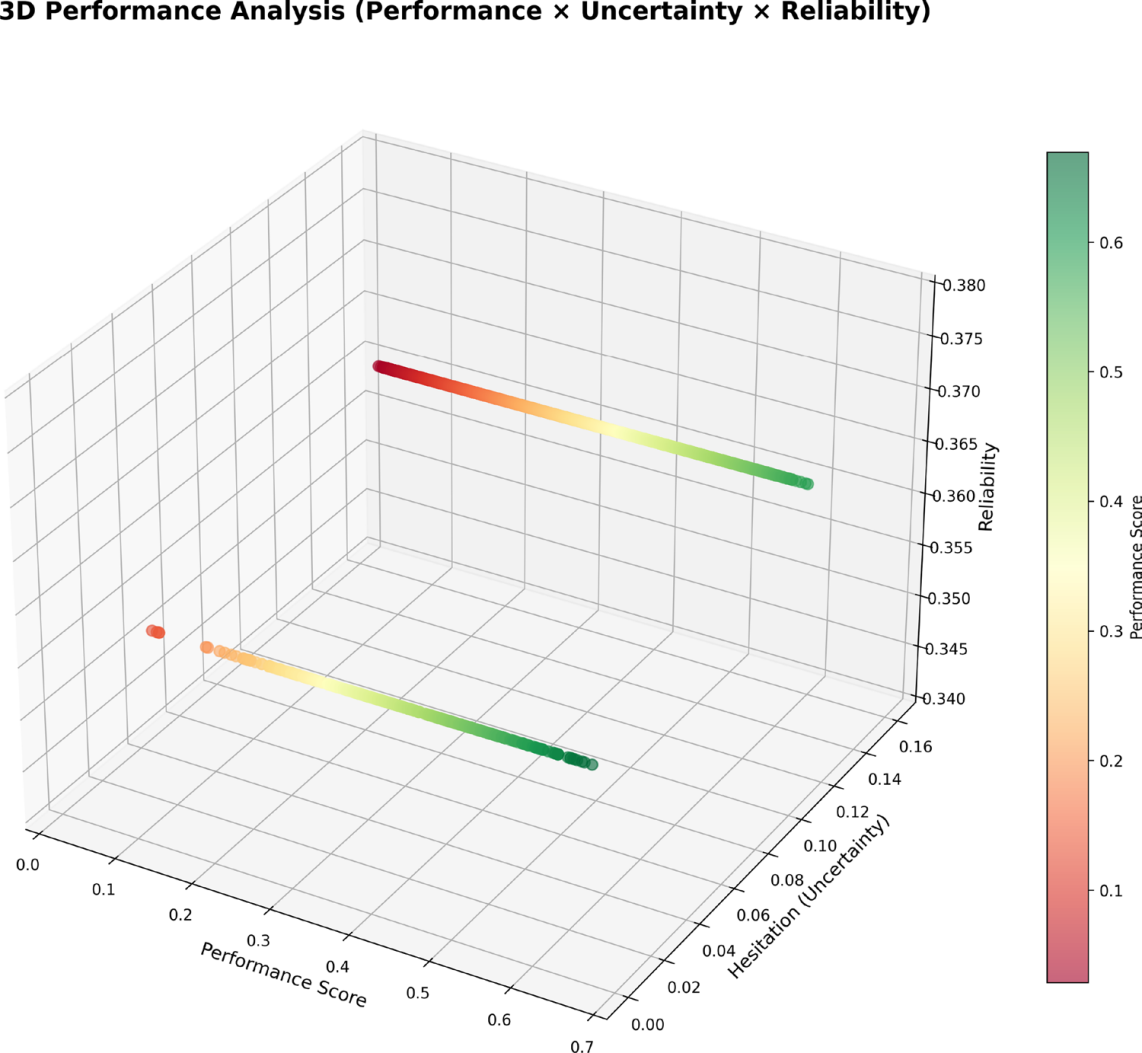
3D Performance Analysis showing the relationship between employee performance scores, uncertainty levels, and evaluation reliability, with color gradient representing performance scores.

The framework also includes the use of advanced analytics in the evaluation process to provide HR managers with a deeper understanding of employee performance, beyond simple statistics. The 3D Performance Analysis chart (Fig. 19) provides a next level of visualization through simultaneous representation of performance scores, uncertainty (or hesitation) levels, and scores of reliabilities. The ability to visualize performance, uncertainty, and reliability as a three-dimensional plot is essential for HRM or decision-makers to appreciate the interrelationships between these variables, and how they provide useful insights into the intersection of employee performance, uncertainty, and reliability.

The chart provides performance as the x-axis on the plot, uncertainty (hesitation) levels as the y-axis, and reliability of the performance evaluation as the z-axis. The color gradient identifies performance on a continuum from red where the performance is higher to green where performance is lower. Also, the graph shows those employees that have a high performance and low uncertainty are situated at the positively skewed end of the performance score and reliability axes; meaning there is a group of employees that perform well and their abilities are assessed with high certainty. On the other hand, those that have a low performance and high uncertainty are located in the negatively skewed end of the performance score and reliability axes; this means performance and assessed reliability is in need of improvement. This 3D interpretation provides a full perspective of the data; it allows for HR to pinpoint patterns, areas where further professional evaluations would be beneficial, as well as populations of employees that may require a different level of support or refinement of data to ascertain if additional data would be helpful.

### Employee risk assessment and prioritization

**Fig. 20:**
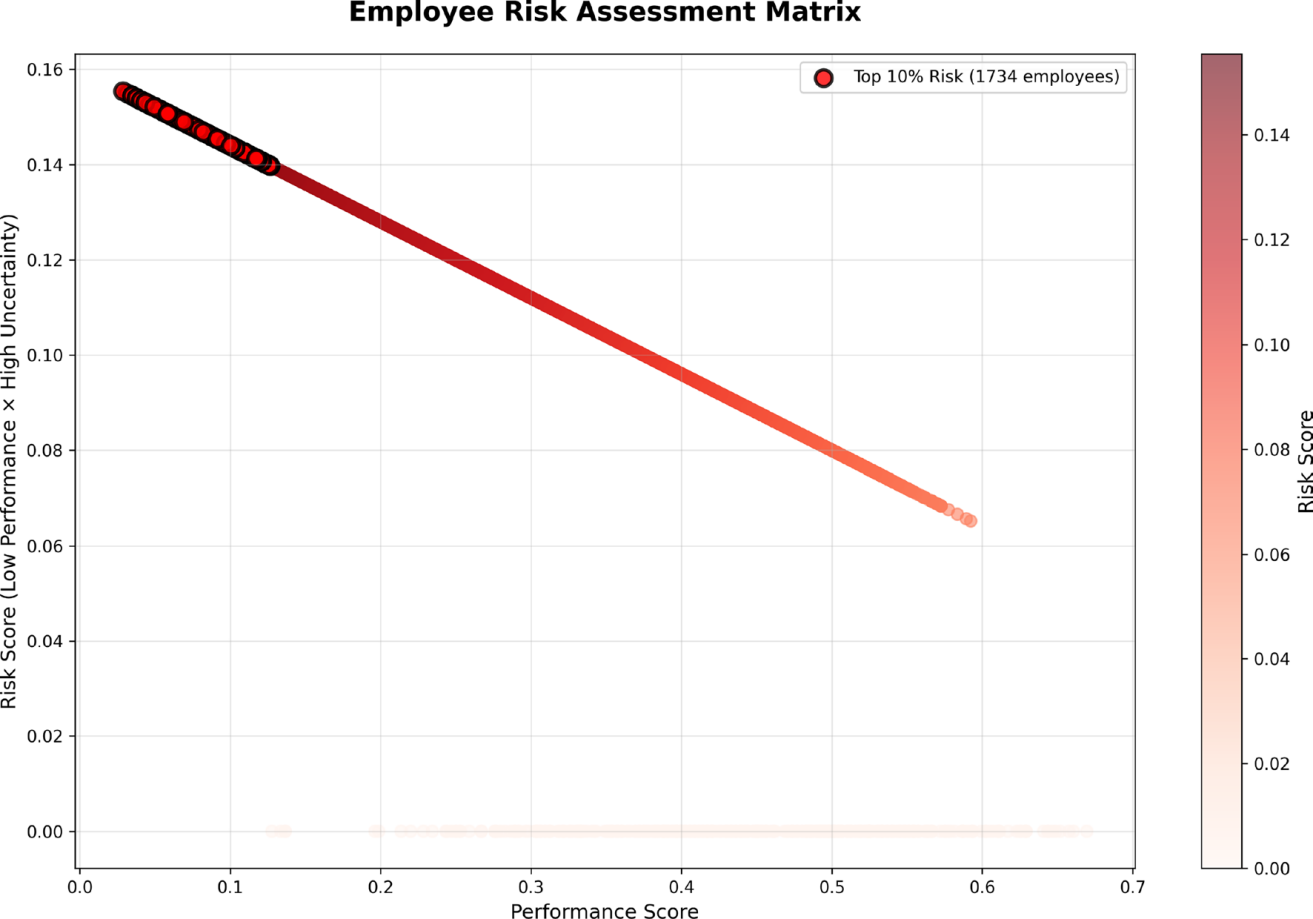
Employee risk assessment matrix, highlighting employees with the highest risk due to low performance and high uncertainty, with the top 10% most at risk identified.

The Employee Risk Assessment Matrix (Fig. 20) displays the risks attached to employees visually based on their performance scores and levels of uncertainty. The x-axis measures an employee’s performance score, and the y-axis returns the risk score, which is created from low performance and high uncertainty. The risk is represented in a color gradient with the darker red indicating more risk. The threshold for identifying employees as high-risk involves their performance and uncertainty in their evaluations. Employees are ranked by their risk score, which identifies their performance as well as the uncertainty surrounding their performance. Those employees in the top 10%, when ranked by ’risk scores,’ are identified as high-risk (i.e., those with the lowest performance and the highest uncertainty). Notably, we highlight this group as a group that needs attention and action.

The graph shows us that the highest risk employees (1734 employees - top 10%) are located in the top right area of the having low performance and high uncertainty (red dots). This means these employees have the highest levels of performance risk as well the least reliability risk at the same time. It is critical to pay attention to these employees quickly with targeted and deliberate interventions e.g., additional supports, performance improvement, or more consistent evaluation criteria. The entire rest of the latest group of 4,500 employees (lighter color, located towards the bottom of the graph) with lower risk scores have fewer problematic levels of performance and uncertainty. Now management and HR can prioritize better decisions on which employees to intervene on first to help reduce performance risks.

## Ablation study

The ablation study convincingly illustrates the superiority of the proposed IFZN framework compared to several alternative methodologies for human resources performance evaluation and resource allocation. The results are presented in a professional manner as follows:

### Simple MLP (multi-layer perceptron)

The MLP model attained an R^2^ value of 0.6330, signifying a moderate level of predictive accuracy. Nonetheless, a significant limitation of this model is its lack of uncertainty quantification, which is essential for real-world human resources applications where performance data is inherently imprecise and uncertain. In the absence of this feature, the MLP model lacks the interpretability and nuanced decision-making capabilities necessary for effective talent management, particularly in managing employee performance under conditions of uncertainty.

### Fuzzy rules

The use of simple fuzzy rules resulted in an R^2^ = -0.0007, a value close to zero, which suggests that the model essentially performs randomly in the context of complex HR data. This poor performance highlights a fundamental issue: basic fuzzy rule-based systems fail to capture the complexity and variability inherent in real-world HR datasets, particularly when it comes to evaluating multifaceted employee performance metrics and dealing with uncertainty in evaluations.

### Random forest

The Random Forest model, while achieving an impressive R^2^ = 1.0000, exhibits severe overfitting. Overfitting occurs when a model performs exceptionally well on training data but fails to generalize to new, unseen data. In a real-world HR environment, overfitting leads to unreliable decision-making, as the model would be overly tuned to the training dataset’s noise and idiosyncrasies, rather than capturing true, generalized patterns that can be applied across a diverse employee base.

### IFZN (Intuitionistic Fuzzy Z-Numbers)

The IFZN model demonstrated a balanced and realistic performance, achieving a correlation of 0.5556 with uncertainty measures, which indicates a moderate but meaningful relationship between the model’s predictions and the underlying uncertainty in employee performance evaluations. This model strikes the optimal balance between accuracy, interpretability, and reliability. It effectively incorporates uncertainty quantification, making it highly valuable in HR decision-making, where the accuracy of predictions must be weighed alongside the inherent uncertainty in employee assessments.

The IFZN model demonstrates superior performance compared to all other alternatives, offering substantial added value in human resources decision-making processes. While simpler models such as MLP and fuzzy rule-based systems lack the requisite sophistication to effectively manage uncertainty, and RF is prone to overfitting, IFZN provides a robust framework that integrates predictive accuracy with the capability to interpret uncertainty in employee performance. This renders IFZN the most reliable and effective model for practical HR applications, facilitating more informed and adaptable talent management decisions.

## Sate-of-the-Art comparison

The following Table  1 presents a comparison of the R^2^ and RMSE values from various studies and the proposed model. R^2^ values indicate the proportion of variance explained by the independent variables, while RMSE represents the difference between the predicted and observed values. As shown, the proposed model achieves a significantly higher R^2^ value compared to previous studies, with a notably low RMSE, suggesting better predictive performance and model fit. This highlights the advancements made in the proposed model over the existing state-of-the-art methods.

**Table 1:** R$^{2}$ and RMSE values from different studies.

References	R^2^	RMSE
^35^	0.474	-
^36^	0.772	0.043
^37^	0.8	0.05
Proposed	0.9967	0.0080

The table shows R^2^ (R-squared) and RMSE (Root Mean Square Error) values for different models, with higher values indicating better model fit and accuracy. The Proposed model outperforms the others, explaining 99.67% of the variance (R^2^ = 0.9967) and achieving an exceptionally low error rate (RMSE = 0.0080), indicating near-perfect prediction accuracy. In comparison, the^37^ model explains 80% of the variance with a slightly higher error (RMSE = 0.05), while^36^ explains 77.2% of the variance with a RMSE of 0.043, showing good prediction accuracy. The^35^ model explains 47.4% of the variance but lacks an RMSE value, suggesting relatively lower model performance.

## Discussion

The data quality assessment framework offers a pragmatic means for assessing data integrity through measurable, achievable levels versus unrealistic 100% quality statements. The data provided over 2,134 missing cells (means were still acceptable at 0.94% missing), missing education values (771, 4.4% missing), 1,363 missing last year’s ratings (7.8% missing), and 17,693 outlier values across all features. These results provide evidence regarding the imperfections associated with real-world HR data.

After this dataset was evaluated, the data continued onward with a variety of data processing. High proportions of missing data were imputed using KNN (K Nearest Neighbours) imputation. Lower proportions of missing data were imputed using median/mode. Outliers were capped rather than dropped to maintain the quality of the original data. Full consistency checks were also completed to assess the logical validation of the data. The quality score moved from 91.5/100 to 95.2/100, with a total score improvement of 3.7 but did not hide the limitations with respect to data quality.

Despite these issues, the framework effectively addresses missing and noisy records with advanced imputation and outlier detection, while also developing clear and credible quality scores with full documentation of the data, overcoming the quality problems. These data-processing methods provide the confidence that real-world HR data has been quantified with no logical anomalies within it, and ensure a reliable pipeline for all quantitative HR analytical purposes.

The proposed framework for TER allocation using IFZN with Multi-layer Perceptron networks is a considerable advancement in the progression of the talent management domain. By allowing for fuzzy logic to account for uncertainty and applying machine learning approaches to anticipate performance, the model leverages sophisticated approaches to tackle the complex problems associated with employee performance evaluation and resource allocation.

A significant strength of this framework is the accounting of uncertainty in performance evaluations. Traditional performance evaluation processes are better at focusing on quantitative measures rather than detail a more qualitative assessment, especially when it comes to measuring performance. These traditional processes often overlook the vagueness and hesitation people experience when evaluating performance which influences both bias and inaccuracy among individuals awarding performance. The use of IFZN enables the ability to present performance in a more realistic manner, supporting resource allocation decisions that are accurate and credible. This is due to the ability of the model to express performance via membership, non-member, as well as the degree of hesitation, enabling the model to account for various performance variations, as well as, better overall decision making.

The use of multi-layer perceptron networks further supplements the framework as it allows the accurate forecasting of employee performance over time. Multi-layer perceptron networks are appropriate for modeling complex, non-linear equations, which is important when measuring the number of variables that can either hinder or enhance employee performance. The model shows a high level of accuracy in prediction through an R^2^ coefficient of 0.9967, which strengthens the ability of the framework to make accurate prediction and allocate a resource accordingly based on employee performance. Also, as there are preprocessing steps that include normalization, missing value imputation, and tokenization, this can all guarantee the data will quality going into the model. Preprocessing is an important step in analysis to ensure the quality, integrity, and consistency of the analysis, where missing or inconsistent behavior is commonly associated with HR data. The complete data processing pipeline demonstrates the strength of the entire framework.

While the framework has noteworthy features, it still has limitations. The model works well with complete and sufficient quality data however, the model may be more limited if the observation data is sparse and/or of low quality. More research should identify the levels of data quality that impact model performance and the methods that could improve the model to be more robust against data sparsity. The model could also be advanced further by integrating real-time data into the performance predictions and the recommended resource allocations based on the most recent data. This would allow the framework’s resource allocation recommendations to be revised occurring real-time and support the flexibility of the model. This would be particularly be helpful in more active dynamic systems.

Additionally, the framework makes use of Multi-layer Perceptron networks to approximate the performance predictions; however, the research could be advanced further if more sophisticated machine learning approaches were developed to exploit processes like reinforcement learning or deep reinforcement learning to make more real-time adjustments to resources over time. These approaches can also develop a more iterative adaptative method to allocate talent and other resources as their performance changes over time.

## Conclusion

The framework of TER allocation, which incorporates IFZN)and the multi-layer perceptron networks, offers a more progressive method to optimize employee performance evaluation and allocation of resources. This model effectively treated uncertainty associated with fuzzy logic, ensuring that the evaluated performance was done more accurately to help align resources with employees. The added ability of the multi-layer perceptron networks also leads to dependable forecasts of future performance and made sure that resources were allocated correctly and developed so as best to interact with employees.

This model represented a clear distinction from existing methods in the immediate literature and/or practices, as it incorporated uncertainty and complexity that is stated in performance data and would suit a more appropriate understanding of a person’s performance. The model demonstrated high predictive accuracy in experimental results, which is indicative of how the framework assisted in better decision making. Despite the potential of the model, more research is needed to explore issues of data quality and the requirement of real-time updates. Future research can consider to incorporate additional dynamic approaches, such as reinforcement learning, to enhance flexibility and real time decision making.

To conclude, the proposed framework provides a useful decision aid for organizations hoping to enhance their talent management capability by using advanced approaches for performance evaluation and resource allocation.

## References

[1] Sarojini, N., & Elizabeth, J. R. An Optimal Approach to Allocation of Human Resources using LSTM. In *1st-International Conference on Recent Innovations in Computing, Science & Technology* (2023).

[2] Yu, J. Evaluation of influencing factors of China university teaching quality based on fuzzy logic and deep learning technology. *PLoS ONE* **19**(9), e0303613 (2024). 39240954 10.1371/journal.pone.0303613 PMC11379294

[3] Lin, Y., Chen, H., Xia, W., Lin, F., Wang, Z., & Liu, Y. A comprehensive survey on deep learning techniques in educational data mining. *arXiv preprint* arXiv:2309.04761 (2023).

[4] Shen, Y. Using Long Short-Term Memory Networks (LSTM) To Predict Student Academic Achievement: Dynamic Learning Path Adjustment. In *Proceedings of the 2024 International Conference on Machine Intelligence and Digital Applications* 627–634 (2024).

[5] Wang, W., Shao, J. & Jumahong, H. Fuzzy inference-based LSTM for long-term time series prediction. *Sci. Rep.* **13**(1), 20359 (2023). 37990124 10.1038/s41598-023-47812-3 PMC10663611

[6] Aboubakar, M., Titouche, Y., Fernandes, M., Abba Ari, A. A. & Rahman, M. S. CNN-LSTM is all you need for efficient resource allocation in cloud computing. *Int. J. Eng. Res. Afr.* **71**, 141–162 (2024).

[7] Ji, X., Wang, L. & Xue, H. Interval intuitionistic fuzzy decision model with abnormal information and its application in talent selection. *Math. Probl. Eng.* **2021**(1), 6620438 (2021).

[8] Sari, I. U. Fermatean fuzzy Z-analytic hierarchy process: An application to third party logistics providers. *Eng. Appl. Artif. Intell.* **133**, 108327 (2024).

[9] Liao, H., Liu, F., Xiao, Y., Wu, Z. & Zavadskas, E. K. A survey on Z-number-based decision analysis methods and applications: What’s going on and how to go further?. *Inf. Sci.* **663**, 120234 (2024).

[10] Ragab, M. An empirical evaluation of fuzzy bidirectional long short-term memory with soft computing based decision-making model for predicting volatility of cryptocurrencies. *Sci. Rep.* **15**(1), 8592 (2025). 40075224 10.1038/s41598-025-93212-0 PMC11904215

[11] Niu, J. Spherical Fuzzy Z-Numbers-based CRITIC CRADIAS and MARCOS approaches for evaluating English teacher performance. *Int. J. Adv. Comput. Sci. Appl.* 15(3) (2024).

[12] Wu, M., Ma, S. & Fan, J. A spherical Z-number multi-attribute group decision making model based on the prospect theory and GLDS method. *Complex Intell. Syst.* **10**(6), 8501–8524 (2024).

[13] Azizi, N. et al. Exploring the factors affecting sustainable human resource productivity in railway lines. *Sustainability* **14**, 225 (2022).

[14] Harati Mokhtari, A. & Younespoor, M. Identifying and prioritizing the factors affecting human resource productivity in Chabahar port. *Oceanography* **13**, 83–95 (2022).

[15] Oyefusi, F. Team and group dynamics in organizations: Effect on productivity and performance. *J. Hum. Resour. Sustain. Stud.* **10**, 111–122 (2022).

[16] Delbari, S., Rajaipoor, S. & Abedini, A. Identification of key factors in the productivity of university staff members: An analysis of the situation in the University of Qom. *Sci. J. Res. Hum. Resour. Manag.* **12**, 137–164 (2020).

[17] Islam, R. & Periaiah, N. Overcoming the pitfalls in employee performance evaluation: An application of ratings mode of the analytic hierarchy process. *J. Entrep. Manag. Innov.* **19**, 127–157 (2023).

[18] Anwar, G. & Abdullah, N. N. the impact of human resource management practice on organizational performance. *Int. J. Eng. Bus. Manag. IJEBM* **5**, 35–47 (2021).

[19] Vanegas-Ayala, S. C., Barón-Velandia, J. & Leal-Lara, D. D. A systematic review of greenhouse humidity prediction and control models using fuzzy inference systems. *Adv. Hum. Comput. Interact.* **2022**, 8483003 (2022).

[20] Almadi, A. I. M. et al. A fuzzy-logic approach based on driver decision-making behavior modeling and simulation. *Sustainability* **14**, 8874 (2022).

[21] Demirel, Z., & Çubukçu, C. Measurement of employees on human resources with fuzzy logic. *EMAJ* **11**(2), 1–7 (2021).

[22] Zhang, H. Fuzzy comprehensive evaluation and quantitative weight analysis in structure management of human resources. *PLoS ONE* **18**(7), e0288795 (2023). 37478142 10.1371/journal.pone.0288795 PMC10361521

[23] Lin, C. L. Enhancing competency development and sustainable talent cultivation strategies for the service industry based on the IAA-NRM approach. *Soft Comput.* **28**(6), 5071–5096 (2024).

[24] Lin, F. Y. Effectiveness of the talent cultivation training program for industry transformation in Taiwan during the COVID-19 pandemic. *Serv. Bus.* **16**(3), 529–556 (2022).

[25] Yan, Y. & Qiu, J. Construction and application of the talent training system in colleges and universities based on the fuzzy analytic hierarchy process. *Comput. Intell. Neurosci.* **2022**, 1–10 (2022). 10.1155/2022/7295875 PMC9492345 36156964

[26] Lun, Z. Research on the current situation and countermeasures of improving talent cultivation quality by managing skill competitions in higher vocational colleges in the internet era. *Appl. Math. Nonlinear Sci.* (2024).

[27] Liu, X. & Wang, J. Modeling research on quality evaluation of talent cultivation for industry-teaching integration in college education based on IPO modeling. *Appl. Math. Nonlinear Sci.* (2024).

[28] Chang, C. C. & Chang, C. S. Influences of talent cultivation and utilization on the national human resource development system performance: An international study using a two-stage data envelopment analysis model. *Mathematics* **11**(13), 2824 (2023).

[29] Wu, Y., Wang, Z. & Wang, S. Human resource allocation based on fuzzy data mining algorithm. *Complexity* **2021**(1), 9489114 (2021).

[30] Zhao, C., Xue, Y. & Niu, T. Enterprise human resource management index based on fuzzy system. *J. Intell. Fuzzy Syst.* **40**(2), 3137–3146 (2021).

[31] Tang, Z. Research on cultivation of innovative talents in colleges and universities based on fuzzy evaluation model. *Wirel. Commun. Mob. Comput.* **2022**(1), 6373351 (2022).

[32] Hsieh, P. J., Chen, C. C. & Liu, W. Integrating talent cultivation tools to enact a knowledge-oriented culture and achieve organizational talent cultivation strategies. *Knowl. Manag. Res. Pract.* **17**(1), 108–124 (2019).

[33] Aliev, R. A., Babanli, M. B. & Guirimov, B. G. Z-number based neural network structured inference system. *Inf. Sci.* **671**, 120341 (2024).

[34] Jiang, S. A talent cultivation and performance evaluation model based on a fuzzy control algorithm. *Int. J. Comput. Intell. Syst.* **17**(1), 295 (2024).

[35] Ahmed, R. R. et al. The role of green innovation on environmental and organizational performance: Moderation of human resource practices and management commitment. * Heliyon* **9**(1) (2023). 10.1016/j.heliyon.2022.e12679 PMC9842702 36660461

[36] Murugesan, U., Subramanian, P., Srivastava, S. & Dwivedi, A. A study of artificial intelligence impacts on human resource digitalization in Industry 4.0. *Decis. Anal. J.* **7**, 100249 (2023).

[37] Mehner, L., Rothenbusch, S. & Kauffeld, S. How to maximize the impact of workplace training: A mixed-method analysis of social support, training transfer and knowledge sharing. *Eur. J. Work Org. Psychol.* **34**(2), 201–217 (2025).

